# Macrophage/Epithelium Cross-Talk Regulates Cell Cycle Progression and Migration in Pancreatic Progenitors

**DOI:** 10.1371/journal.pone.0089492

**Published:** 2014-02-19

**Authors:** Kristin Mussar, Andrew Tucker, Linsey McLennan, Addie Gearhart, Antonio J. Jimenez-Caliani, Vincenzo Cirulli, Laura Crisa

**Affiliations:** Department of Medicine, Institute of Stem Cell and Regenerative Medicine, University of Washington, Seattle, Washington, United States of America; University of Bremen, Germany

## Abstract

Macrophages populate the mesenchymal compartment of all organs during embryogenesis and have been shown to support tissue organogenesis and regeneration by regulating remodeling of the extracellular microenvironment. Whether this mesenchymal component can also dictate select developmental decisions in epithelia is unknown. Here, using the embryonic pancreatic epithelium as model system, we show that macrophages drive the epithelium to execute two developmentally important choices, i.e. the exit from cell cycle and the acquisition of a migratory phenotype. We demonstrate that these developmental decisions are effectively imparted by macrophages activated toward an M2 fetal-like functional state, and involve modulation of the adhesion receptor NCAM and an uncommon “paired-less” isoform of the transcription factor PAX6 in the epithelium. Over-expression of this PAX6 variant in pancreatic epithelia controls both cell motility and cell cycle progression in a gene-dosage dependent fashion. Importantly, induction of these phenotypes in embryonic pancreatic transplants by M2 macrophages *in vivo* is associated with an increased frequency of endocrine-committed cells emerging from ductal progenitor pools. These results identify M2 macrophages as key effectors capable of coordinating epithelial cell cycle withdrawal and cell migration, two events critical to pancreatic progenitors' delamination and progression toward their differentiated fates.

## Introduction

The cross-talk between the mesenchyme and epithelia is critical to both the growth of epithelial organs during development as well as tissue regenerative processes in post-natal life [1]. The identification of the cellular mediators of these effects is subject of substantial research efforts in the field of regenerative medicine, as it may lead to uncover signals that could be targeted to restore loss of tissue function in degenerative diseases, organ autoimmunity or injury.

Among mesenchymal cell types that dynamically populate both developing and injured tissues are cells of the innate immune system, namely macrophages. Hence, high numbers of macrophages colonize virtually all epithelial tissues early in embryogenesis [2], and key trophic effects of this immune cell subset have been inferred by the severely impaired growth of epithelial organs displayed by animal models deficient in macrophages or macrophage-dependent functions [3]–[7]. Recruitment of myeloid cell populations from the bone marrow to the periphery continues to be essential in adulthood for the maintenance of tissue integrity, since, in their absence, tissue repair and regenerative events following injury are critically blunted [8]–[13]. To date, experimental evidence indicate that macrophages may primarily influence the growth and/or regeneration of epithelial organs indirectly, i.e. by supporting functions such as clearance of dying cells [4], [14], angiogenesis [15]–[17] and remodeling of extracellular matrices [18]. Whether macrophages can directly dictate select developmental options in epithelia remains presently unclear.

During pancreatic development, at E14.5–15.5 gestational age, epithelial progenitors emerge from a rudimentary ductal tree through a regulated sequence of events that includes withdrawal from the cell cycle, delamination into the surrounding mesenchyme and differentiation into endocrine or exocrine cell types [19]–[21]. As such, while providing a pool of progenitors competent to execute specific developmental steps and make divergent lineage choices, the E14.5/E15.5 pancreas represents a valuable model to study how such epithelial programs might be impacted on by other exogenous cellular cues. In this regard, *in vivo* and *in vitro* studies have provided evidence that, over-imposed to a hierarchy of transcription factors expressed by the epithelium [19]–[21], interactions of the epithelium with the pancreatic mesenchyme govern the balance between the exocrine and the endocrine developmental fate of progenitors [22], [23] and are required for the growth of the pancreatic epithelial compartment as a whole [24]. At present, few studies have reported the presence of tissue macrophages within the pancreatic mesenchyme and noted reduced growth of endocrine cells in their absence [25], [26]. However, the possible role of macrophages as regulators of select developmental events in the pancreatic epithelium remains unknown.

A corollary to this question is whether diverse states of activation of tissue macrophages differentially affect pancreatic developmental programs. Indeed, macrophages resident within tissues may adopt a spectrum of functional states. At the extreme of this spectrum are classically (M1) and alternatively activated (M2) macrophages [27]. M1 phenotypes are acquired by macrophages upon encounter with pathogens, and lead to the production of high levels of pro-inflammatory mediators and reactive nitrogen intermediates that contribute to pathogen clearance. Conversely, “alternative” or M2 activation states are characterized by the production of lower levels of pro-inflammatory cytokines, synthesis of decoy anti-inflammatory receptors, little or no nitrogen derivatives, as well as production of mediators of tissue remodeling. As such, M2 macrophages have immune-regulatory functions that dampen inflammation and promote repair during wound healing. Stimulation of macrophages with cytokines such as IFNγ/LPS or IL4 polarize them toward M1- and M2-like activation states, respectively [28], [29], providing an approach to study *in vitro* effector functions related to these states.

Interestingly, the M2-like phenotype is the predominant activation state of macrophages resident in embryonic tissues [30]. The M2 phenotype is also induced in response to tissue damage during the resolution phase of wound healing, implying that, during tissue repair, a window of opportunity exists for M2 macrophages to recapitulate, post-natally, fetal-like inductive functions on epithelia. Hence, animal models of wound healing, in which such polarized macrophages are present in high numbers, may provide ideal *in vivo* systems to assess the functional impact of M2 macrophages on developing pancreatic epithelia. In this regard, we have previously described a mouse model in which the tissue microenvironment at site of wound healing is enriched for cells of the macrophage lineage [31]. In these mice, inactivation of Id1 and Id3 genes results in functional defects of vascular and hemopoietic cell lineages, leading to impaired engraftment of tissue transplants. However, reconstitution of Id1+/−Id3−/− mice with wild type bone marrow (BM) rescues the engraftment defects and results in the enhanced recruitment of BM-derived vasculogenic and myeloid cells at sites of tissue repair [31]–[33]. Importantly, using microarray gene expression profiling, we demonstrated that myeloid cells isolated from tissue grafts in BM-reconstituted Id1+/−Id3−/− mice are functionally skewed toward M2 immune responses [31].

In the present study, using both *in vivo* studies in Id1/Id3-deficient mice and *in vitro* organ cultures, we uncover a role of M2 macrophages in instructing the pancreatic epithelium to acquire a motile phenotype and negatively regulate cell cycle progression. Induction of these phenotypes in transplants of embryonic pancreatic epithelium is associated with an increased frequency of cells committed to the endocrine lineage emerging from the ductal pool. We demonstrate that macrophages mediate these effects through modulation of the adhesion receptor NCAM and up-regulation of an uncommon isoform of PAX6 in a subset of ductal cells.

## Matherials and Methods

### Mouse breeding and bone marrow reconstitution

All mice strains were bred and housed at the University of Washington pathogen-free facility. Id1^+/−^Id3^+/−^ heterozygous breeders, Id1^+/−^Id3^−/−^ experimental and WT mice were genotyped as described previously [31]. WT mice were used as bone marrow donors. Bone marrow (BM) cells were flushed from femurs with RPMI-10% FCS, and injected intravenously (5–10×10^6^) in 6- to 8-week-old lethally irradiated WT and Id1^+/−^Id3^−/−^ mice (1,200 rads). Eight to 12 weeks post-BM reconstitution, mice were transplanted with embryonic pancreatic explants. All procedures were approved by the Institutional Animal Care and Use Committee of the University of Washington (Protocol number 4231-01). All surgical procedures were performed under anesthesia and all efforts were undertaken to minimize suffering.

### Embryonic pancreas dissection, collagenase digestion and transplantation of epithelium- enriched fractions

Time-dated pregnant WT females were used as donors of E14.5–E15.5 embryos. Embryonic pancreatic tissue was dissected and digested for 1 hour at 37°C in HBSS/0.1% collagenase A/20 µg/ml DNase. To facilitate dissociation of the mesenchyme from the epithelial component of the pancreas, the tissue was pipetted at 15 minutes intervals during digestion. Following this step, the resulting epithelial clusters were separated from the mesenchymal fraction through 3 rounds of gravity sedimentations on sterile medium. This procedure consistently resulted in >80% enrichment of epithelial cells, as measured by immunostaining and flow cytometric analysis of cell suspensions for the epithelial marker E-cadherin. Epithelial cell clusters were then transplanted as a cell pellet under the kidney capsule of naive, BM-reconstituted Id1^+/−^Id3^−/−^, or WT mice. Each transplant recipient routinely received pancreatic epithelial clusters derived from 5–6 embryos.

### Histology and morphometric analysis

Two weeks post-transplantation, mice were euthanized and kidneys carrying the grafts harvested, fixed in 4% PFA and embedded in OCT for histology. Seven-micron cryostat sections were cut and processed for immunofluorescence. Briefly, after citrate antigen retrieval, sections were permeabilized in 0.05% Triton-X 100, blocked in 50 mM Glycine for 10′ at RT followed by incubation in 1% BSA/2% donkey serum for 1 hour at RT. Primary antibodies used for immunostaining were: mouse anti-E-cadherin (BD Biosciences, Clone36/E-cadherin), goat anti-PDX-1 (kind gift of Dr. M. Sander, UCSD), sheep anti-insulin (The Binding Site), mouse anti-glucagon (clone 79bB10, Sigma), mouse anti-PCNA (clone PC10, Santa Cruz antibodies), rabbit anti-somatostatin (Dako), rabbit anti-PP (Invitrogen), rabbit anti-alpha-amylase (Sigma), rabbit anti-NCAM (kind gift of Dr. K. Crossin, TSRI), rabbit anti-PAX6 and mouse-anti-P27^Kip^ (Chemicon). Secondary antibodies (Jackson Immuno-Research Lab) were species-specific Fab^2^ fragments Rhodamine-Red conjugated donkey anti-mouse IgG, FITC-conjugated donkey anti-goat IgG, Cy5-conjugated donkey anti-sheep IgGs, Cy5 or FITC conjugated-donkey anti-rabbit IgGs. In situ immuno-detection of P27^Kip^ was obtained by tyramide amplification using the TSA kit #2 (Molecular Probes) as per manifacturer's instructions. Apoptotic cells were detected using the ApoTag-Fluorescein in Situ Apoptosis Detection Kit (Millipore) in combination with immunostaining for E-cadherin. After staining, sections were mounted and visualized at a NIKON Eclipse-800 microscope, equipped with a Spot II CCD camera or at a Zeiss Axiovert microscope equipped with a scanning laser confocal attachment (Nikon A1). Morphometric analysis was performed on ∼20–30 sections per graft, collected at 100-µm intervals until exhaustion of the grafts, using the Spot Advanced and ImageProPlus software. Collectively morphometric analysis was performed on a total of 150–240 sections per group.

### RNA isolation and Real-time PCR

RNA was isolated using the RNAeasy Kit (Qiagen), treated with DNase (Ambion) for 30 minutes at 37°C, and purified by phenol/chloroform extraction and ethanol precipitation. Purified RNA was retro-transcribed into cDNA using oligo dT primers and the Superscript III reverse transcriptase (Invitrogen). Expression of specific genes was assayed by either standard semi-quantitative PCR or in triplicates by real time PCR using either the SensiMix SYBR green PCR master mix (Bioline) or the iTaq SYBR green amplification system (Biorad). The following mouse-specific primer pairs were used: *18S* forward GTAACCCGTTGAACCCCATT and reverse CCATCCAATCGGTAGTAGCG; *E-cadherin* forward ACTGTGAAGGGACGGTCAAC and reverse TGTfCCCGGGTATCATCATCT; *PDX-1* forward GAAATCCACCAAAGCTCACG and reverse TTCAACATCACTGCCAGCTC; *Ngn3* forward GAGGCTCAGCTATCCACTGC and reverse TTGGAACTGAGCACTTCGTG; *Neuro-D1* forward GCTCCAGGGGTTATGAGATCG and reverse CTCTGCATTCATGGCTTCAA; *NKX6.1* forward GACGGAGAGTCAGGTCAAGG and reverse AGAGTTGGGTCCAGAGGTT; *NKX2.2* forward TCTACGACAGCAGCGACAAC and reverse TTGTCATTGTCCGGTGACTC; *INS-1* forward CAGCCCTTAGTGACCAGC and reverse GCTCCCCACACACCAGGTA; *Glucagon* forward GATGAACACCAAGAGGAA and reverse TCCAAGTAAGAACTCACAT ; *PAX6* (isof1+5a) forward CAGCTTGGTGGTGTCTTTGT and reverse ACTTGGACGGGAACTGACAC ; *ΔPD-PAX6* forward CAAAACTCTTGACAGGAAGGAGGG and reverse GCCTCAATCTGCTCTTGGGTAA.

Primers for the amplification of human transcripts were: *E-cadherin* forward GAACGCATTGCCACATACAC and reverse ATTCGGGCTTGTTGTCATTC; *PDX-1* forward CCTTTCCCATGGATGAAGTC and reverse GGAACTCCTTCTCCAGCTCT; *NG3* forward CTATTCTTTTGCGCCGGTAG and reverse ACTTCGTCTTCCGAGGCTCT; *NKX2.2* forward GAACCCCTTCTACGACAGCAG and reverse TTGTCATTGTCCGGTGACTC; *NKX6.1* forward ATTCGTTGGGGATGACAGAG and reverse CCGAGTCCTGCTTCTTCTTG; *MAF-B* forward CCTGGCTTTCTGAACTTTGC and reverse ACGTTCTCTATGCGGTTTGG; *Insulin* forward GGGGAACGAGGCTTCTTCTA and reverse CACAATGCCACGCTTCTG; *Glucagon* forward GAATTCATTGCTTGGCTGGT and reverse CGGCCAAGTTCTTCAACAAT; *Somatostatin* forward CACTCTCCAGCTCGGCTTTC and reverse AGTACTTGGCCAGTTCCTGC; *PP* forward CGCTGTCCATCGTCCTGG and reverse GTACTTGGCCAGTTCCTGCT; *NCAM* forward GCTGGACAAAGATGGGGAACA (specific to exon6-exon7 boundary from RefSeq NG-032036.1) and reverse CGCCAGCCTTGTTCTCAGCAAT.

Conditions for standard PCR were: 95°C for 5 minutes, followed by 95°C for 30 seconds, 57°C for 30 seconds and 72°C for 30 seconds for 36 cycles, with a final extension at 72°C for 5 minutes

Real time amplification conditions for mouse primers were 95°C for 15 seconds and 60°C for 45 seconds, for 40 cycles. Conditions for human NCAM real time PCR were 95°C for 15 seconds, 58°C for 30 seconds and 72°C for 20 seconds, for 40 cycles. For each primer combination, amplification efficiency was consistently >95%. Threshold cycle numbers (*Ct*) were determined using the SDS 2.3 software (Applied Biosystems) and analyzed using the ΔΔ*Ct* method. All real time PCR reactions were performed using either the 7900HT Real Time PCR system (Applied Biosystems) or the CFX96 real Time PCR system (Biorad).

### Construction of lenti-viral vectors, virus production and cell transduction

cDNA from E15.5 mouse brain was used as template to amplify an open reading frame of the ΔPD-PAX6 mRNA using the Expand High Fidelity Taq polymerase (Roche) and the following primers: forward AAAACTCTTGACAGGAAGGAG and reverse CCTGAATACCCAACTGCTGT. The resulting blunt-ended product was 3′ A-tailed with *Taq* DNA polymerase, ligated into the pcDNA2.1/TOPO-TA vector (Invitrogen) and sequenced. For expression in mammalian cells, an EcoR1 fragment of the TOPO vector containing the ΔPD-PAX6 insert was cloned into the EcoR1 site of the HIV vector ROVER downstream of a human CMV promoter and upstream of an IRES sequence driving a GFP reporter [34]. This HIV vector was VSV-G pseudotyped by packaging in HEK-293T cells transfected with the transgene plasmid and the packaging plasmids pMD.G (VSV-G), pMDLg/p.RRE (*gag* and *pol*), and pRSV-Rev (*rev*). Viral supernatants were harvested after 48 h, concentrated by ultracentrifugation and viral titers, defined as transducing units/ml or multiplicity of infection (MOI), estimated by transduction of 293T-cells and flow cytometric analysis of GFP^+^ cells. Viral preparations were used to transduce cell lines at either 5 or 30 MOI by overnight incubation in the presence of 5 µg/ml polybrene. Transduction was verified 72 hours later by flow cytometric analysis of GFP^+^ cells and Western blotting analysis of cell lysates for ΔPD-PAX6.

### Macrophage derivation and macrophage/epithelial cell co-cultures

Mouse BM cells, obtained by flushing femurs and tibias, were deprived of endogenous fibroblasts through a 1 hour incubation at 37°C on tissue-culture treated dishes. Non-adherent cells were recovered and cultured for 6 days in RPMI 10% FCS in the presence of M-CSF (20 ng/ml, Peprotech). Purity of macrophage cultures was verified by flow cytometric analysis for F4/80 and Gr1. M1 and M2 macrophage polarization was further induced by culturing the resulting macrophages in the presence of LPS (100 ng/ml) and IFNγ (500 U/ml) (M1) or IL4 (20 ng/ml, Peprotech) (M2) for 48 hours.

For epithelium/macrophages co-cultures, epithelial cell clusters isolated from embryonic pancreata as described above were seeded into V-shaped 96 well plates in 200 microliters of RPMI-5% FCS-5×10^−5^ M β-mercaptoethanol. In experiments using sorted cell subsets, cell clusters were dissociated into single cell suspensions using non-enzymatic dissociation medium (Sigma), labeled with anti-E-cadherin antibody and sorted by flow cytometry according to the expression levels of E-cadherin, using a FACS Aria cell sorter (Beckton Dickinson). Cell equivalents of one embryonic pancreas were seeded per well; 50×10^3^ M1 or M2 macrophages were then added to each well and cultures incubated for 72 hours at 37°C in a humidified incubator.

### Cell lines, flow cytometry and migration assays

The pancreatic ductal lines SU-86 (ATCC), G3LC (a sub-clone of SU.86 derived in our laboratory), and PANC-1 (ATCC) were maintained in RPMI-10% FCS-22 mM glucose, 100 mM pen/strep. For co-cultures with polarized macrophages, 0.5×10^6^ epithelial cells were seeded into a 10 cm dish in growth medium. Macrophages (1×10^6^) were added 8 hours later, and cultures continued over a 48-hour period. Cells were then harvested, labeled with PERCP-conjugated anti-mouse CD45 and FITC-conjugated anti-NCAM Ab and sorted by flow cytometry as CD45^−^NCAM^+^ and CD45^−^NCAM^−^ subsets. For quantification of DNA content, epithelial cell cultures that had reached 50—60% confluency were pulsed for 6 hours with BrDU (10 µg/ml), harvested by trypsinization and fixed in 70% cold ethanol over-night at 4°C followed by incubation with 50 µg/ml RNase A at 37°C for 30 min. After digestion with DNase (Ambion) for 1 hour at 37C and immunostaining with PE-conjugated anti-BRDU antibody (clone Bu20a, Biolegend), cells were labeled with the dye 7AAD and DNA content analyzed at a FACScalibur flow cytometer (Becton Dickinson) using the Flow JO software.

Cell migration was assayed using a two-chamber Trans-well system (24 well plate format, Costar). In preliminary experiments we established that Collagen IV-coated (5 µg/ml) 5 µm-pore filters and Matrigel-coated (1∶30 dilution) 8 µm-pore filters provided optimal adhesive substrates and migration conditions for G3LC and SU86 cells, respectively. For cell migration, G3LC and SU86 cell lines were serum-starved for 6 hours in RPMI-0.5% BSA, trypsinized and seeded in duplicates on the top chamber of the Trans-wells at 40,000 per well in M199/0.5% BSA/4 mM MnCl_2_. Six hundred microliters of the same medium containing 20% FCS or HGF/SF (100 ng/ml) was then placed in the bottom chamber as chemoattractant. Trans-wells were incubated at 37°C for either 3 hours (G3LC) or 5 hours (SU86). Cultures were fixed in 4% PFA, and stained with a solution of 1% Crystal Violet/10 mM Borate/10%ethanol for 30 minutes. After removing the cells from the top surface of the filters with a cotton swab, cells that had migrated to the bottom side of the membrane were visualized at a light microscope (Olympus). Migrating cells were counted in 5 consecutive fields across the Trans-wells using a 10× objective.

### Western Blotting

Cell cytoplasmic and nuclear extracts were prepared using the NE-PER cell extraction Kit (Pierce) in the presence of a proteases inhibitors cocktail (Complete, Roche), and PMSF (1 mM). Protein concentration of lysates was determined by the BCA protein assay (Pierce). Equal amounts of protein (5–10 µg) were then separated under reducing conditions onto 4–12% polyacrilamide gels (Nu-Page, Invitrogen) and transferred by Western blotting onto PVDF membranes (Immobilon, Millipore). After blocking of the membranes in 1% dry milk −0.1% Tween-20 over-night at 4C, the membranes were probed with rabbit anti-PAX6 Ab (Millipore), anti-p21*^Cip^* (SC-397, Santa Cruz antibodies), anti-p27*^kip^* (clone DCS7, Calbiochem), anti-Retinoblastoma, anti-cyclin E (Cell Signaling) or anti-β-actin mAb (clone AC-15, Sigma) followed by HRP-conjugated donkey-anti rabbit or donkey-anti-mouse secondary antibodies. Membrane-bound antibodies were detected by chemoluminescence using an ECL-detection kit (Amersham).

### Statistical Analysis

Statistical significance of differences observed in experiments of tissue morphometric analysis, real time PCR experiments, and migration assays, was validated by Student T-test or by Anova one-group variance test, using the Statview Software with limit for significance set at p<0.05.

## Results

### Developmental effects of tissue microenvironments enriched in M2 macrophages on the embryonic pancreatic epithelium

**Figure 1:**
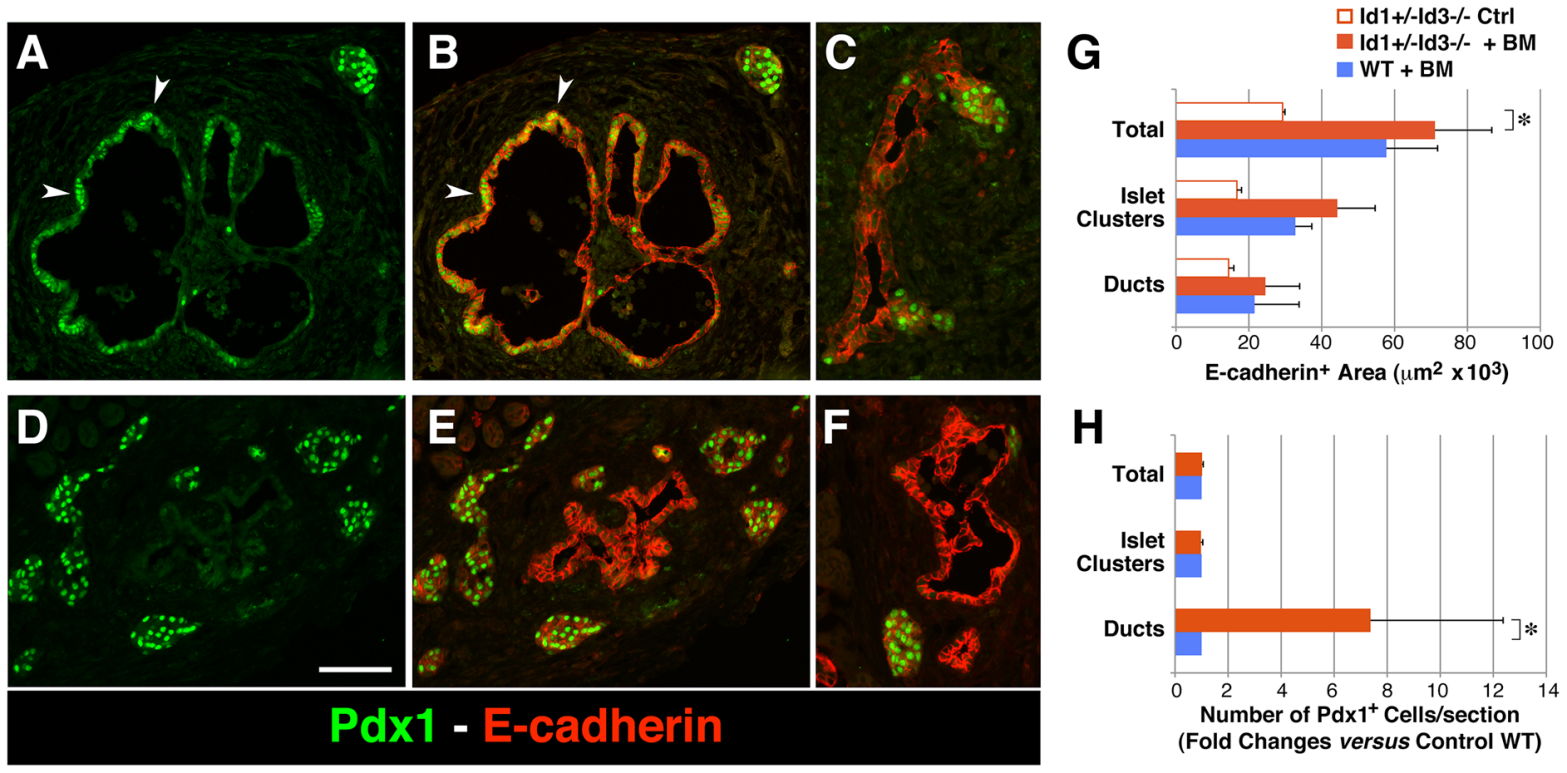
Increased frequency of PDX-1^+^ pancreatic progenitors emerging from the ducts in transplant microenvironments enriched in M2 macrophages. Tissue sections of E14–15.5 pancreatic epithelial grafts transplanted in BM-reconstituted Id1+/−Id3−/− mice (A–C) or WT controls (D–F), stained by two-color immuno-fluorescence for PDX-1 (green) and E-cadherin (red). Numerous PDX-1^+^ cells are comprised within or emerge from ductal structures in the grafts of BM-reconstituted Id1+/−Id3−/− mice (arrowheads). (G–H) Morphometric analysis of E-Cadherin^+^ grafts areas (G) and PDX-1^+^ cells counted within the grafts (H). Bars represent mean ± SEM of n = 4 experiments including a total of 8 grafts in Id1+/−Id3−/− and 6 grafts in WT mice. Scale bar = 60 µm; * *p*≤0.001.

To investigate possible developmental effects of M2-polarized macrophages on the embryonic pancreatic epithelium, explants of embryonic pancreata at E14.5–E15.5 were depleted of the fetal mesenchymal component by collagenase digestion followed by >80% enrichment of epithelial cell clusters by gravity sedimentation. Epithelial clusters were then transplanted under the kidney capsule of BM-reconstituted Id1+/−Id3−/− mice or wild type (WT) controls. Two weeks after transplantation, the grafts were harvested and processed for histology. Immunostaining for E-cadherin revealed the development of both ducts and islet-like clusters within the grafts of either BM-reconstituted Id1+/−Id3−/− mice (Fig. 1A–C) or WT mice (Fig. 1D–F). Morphometric analysis of tissue sections collected throughout the grafts of either hosts showed no significant differences in frequency of apoptotic cells (Figure S1), total E-cadherin^+^ area, or in the E-cadherin^+^ areas of either ductal or islet-like clusters (Fig. 1G), indicating no overt differential effects of either transplant microenvironments on the engraftment of the pancreatic epithelium as a whole. Total E-cadherin+ areas of the grafted tissues were however significantly lower in non-irradiated, non-BM reconstituted naive Id1+/−Id3−/− controls, as compared to BM-reconstituted mice (Fig. 1G) demonstrating the requirement for reconstitution with WT BM for tissue engraftment, as previously described [31].

**Figure 2:**
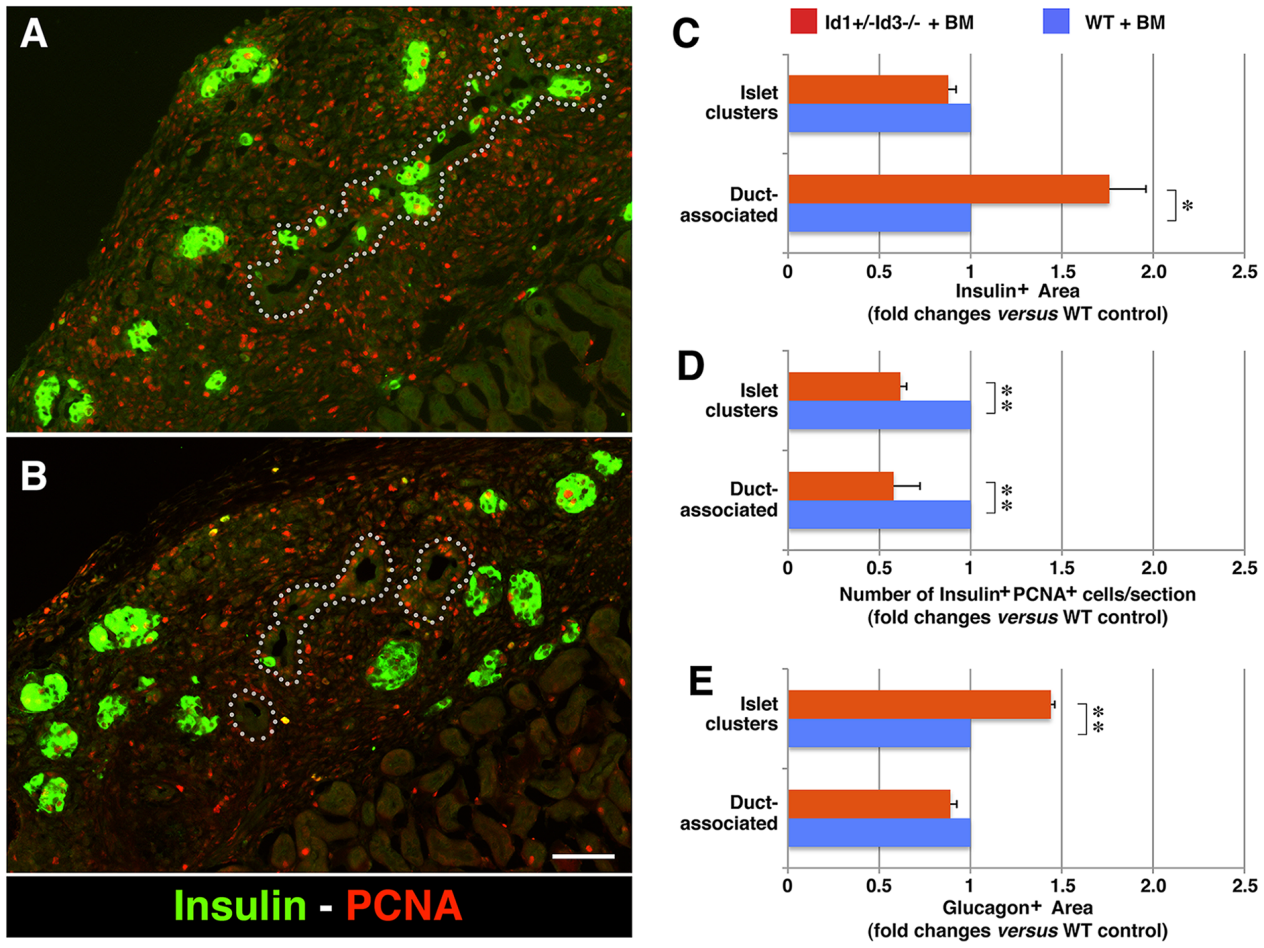
Frequency and proliferative potential of endocrine-committed progenitors in pancreatic transplants. (A–B) Tissue sections of E14–15.5 pancreatic epithelial grafts transplanted in BM-reconstituted Id1+/−Id3−/− mice (A) or WT controls (B), stained by two-color immuno-fluorescence for Insulin (green) and the proliferation marker PCNA (red). Dotted lines delineate boundaries of ductal structures. (C–E) Morphometric analysis of Insulin^+^ areas (C), proliferating Insulin^+^PCNA^+^ cells (D) and Glucagon^+^ areas (E), assessed in the grafts. Bars represent mean ± SEM of n = 3 experiments, including a total of 7 grafts in Id1+/−Id3−/− and 5 grafts in WT mice. Scale bar = 120 µm; *p = 0.01 and ** *p*<0.002.

**Figure 3:**
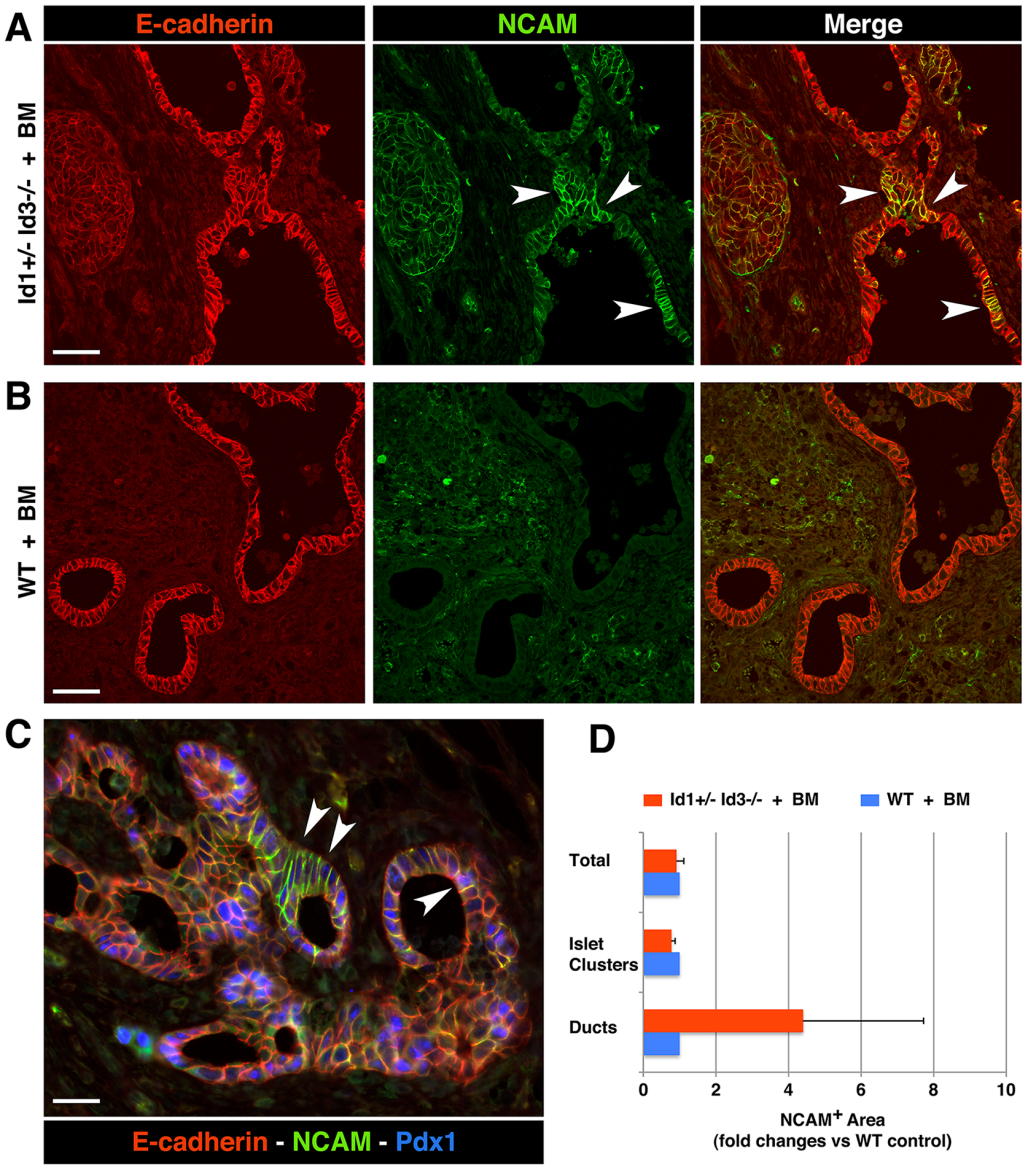
Modulation of NCAM expression in the ductal epithelium of embryonic pancreatic transplants. (A–B) Tissue sections of E14–15.5 pancreatic grafts transplanted in BM-reconstituted Id1+/−Id3−/− mice (A) or WT (B) hosts, immuno-stained for NCAM (green) and E-cadherin (red). Numerous patches of NCAM^+^ epithelial cells are present in ductal structures of grafts from Id1+/−Id3−/− mice (arrowheads) but not in those from WT hosts. C) NCAM^+^E-cadherin^+^ ductal domains comprise PDX-1^+^ progenitors (blue, arrowheads). D) Morphometric analysis of NCAM^+^ areas measured in the grafts. Bars represent mean ± SEM of n = 3 experiments, including a total of 7 grafts in Id1+/−Id3−/− and 5 grafts in WT mice. Scale bars = 90 µm in A–B and 45 µm in C.

Interestingly, two-color immunostaining for PDX-1 and E-cadherin revealed a significant increase in the number of PDX-1^+^E-cadherin^+^ cells associated with ductal structures in the grafts from BM-reconstituted Id1+/−Id3−/− mice (Fig. 1A–C and 1H) as compared to WT transplant recipients (Fig. 1D–H). Additional immunostaining for insulin demonstrated an increased frequency of insulin^+^ cells associated with the ducts in the grafts of BM-reconstituted Id1+/−Id3−/− mice (Fig. 2A and 2C), whereas in WT grafts most insulin^+^ areas appeared to be organized into islet-like clusters away from the ducts (Fig. 2B). Further immunostaining for markers of cell proliferation demonstrated a ∼40% decrease in the number of insulin^+^PCNA^+^ cells within islet clusters and ducts (Fig. 2D) in the grafts of BM-reconstituted Id1+/−Id3−/− mice, indicating decreased proliferation of ß-cells. Lastly, glucagon^+^ areas were increased of ∼1.5 folds in the islet clusters engrafted in BM-reconstituted Id1+/−Id3−/− mice, (Fig. 2E), whereas glucagon^+^ cells associated with the ducts where rare and did not differ from that observed in WT grafts. No significant differences in the frequency of other endocrine cell types (e.g. PP, somatostatin, ghrelin cells) were observed (not shown). In addition, consistent with the prevalent endocrine over exocrine development previously observed in embryonic pancreatic explants depleted of fetal mesenchymal cells [22], [23], no exocrine amylase^+^ developed in the grafts of either hosts (not shown). However, one additional phenotype that we noted in the grafts of BM-reconstituted Id1+/−Id3−/− mice was an increased frequency of duct-associated NCAM^+^ cells (Fig. 3A–B arrowheads, and D), many of which expressed PDX-1 (Fig. 3C).

These results indicate that transplant environments enriched for M2-polarized macrophages support the accumulation of PDX-1^+^, NCAM^+^ and insulin-producing endocrine cells lining within and/or adjacent to the ductal epithelium. In light of the evidence that induction and sustained expression of NCAM are associated with differentiation of endocrine cell lineages [35], [36], and that PDX-1 and insulin expression in duct-associated cells occurs in models of islet neogenesis, [37]–[42], our results suggest that M2 macrophage-enriched microenvironments support the recruitment of islet progenitors from ductal domains. However, as insulin^+^ cells emerge from the ductal compartment and organize into islet-like clusters away from the ducts, this environment restricts their proliferative capability.

### M2 macrophages promote NCAM expression and an uncommon PAX6 transcriptional phenotype in embryonic pancreatic organ cultures

**Figure 4:**
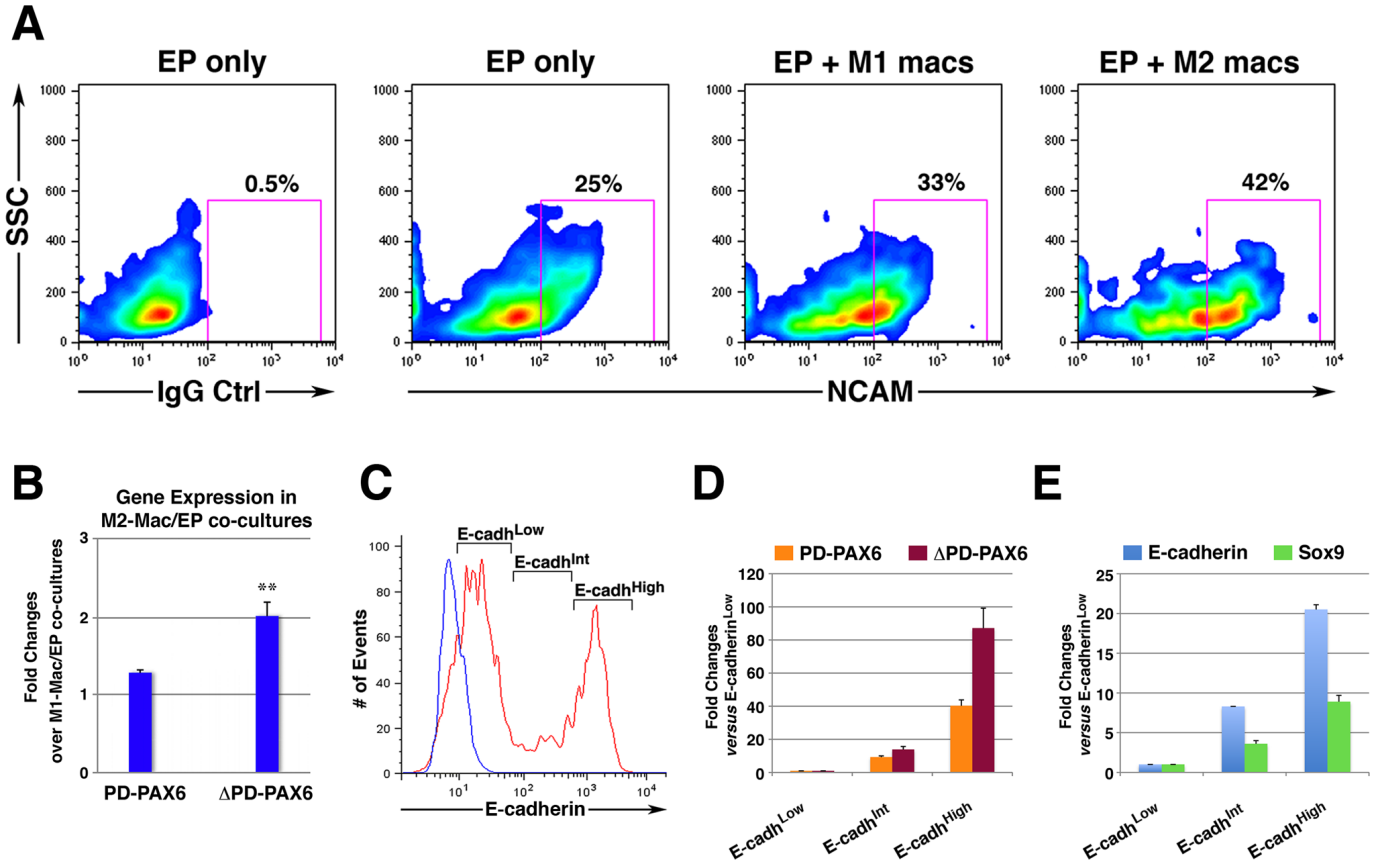
Differential induction of NCAM and PAX6 by M2 versus M1 macrophages in organ cultures, and in select pancreatic epithelial subpopulations. A) E14–15.5 pancreatic epithelial cells, cultured for 3 days in medium alone (Ep) or in the presence of M1- (Ep+M1 macs) or M2- (Ep+M2 macs) macrophages, were immuno-stained for NCAM and the pan-leukocyte marker CD45. Flow cytometric contour plots of cells gated as CD45 negative (i.e. epithelial cells only) are shown, and percentages of NCAM^+^ cells are indicated in the right quadrants. Representative of n = 4 experiments. B) qPCR analysis of the indicated gene transcripts detected in macrophage/embryonic epithelium organ cultures. Values from each experimental condition were normalized to E-cadherin mRNA. Bars are mean ± SEM of n = 4 experiments; ** *p*<0.01. C) Flow cytometric hystogram of epithelial cells from E14–E15.5 pancreata stained for E-cadherin (red) or IgG control (blue). The sorting windows used for the isolation of E-cadherin High, Intermediate and Low populations are shown. (D–E) qPCR analysis of E-cadherin, SOX9 and PAX6-specific transcripts expressed in the indicated sorted populations. Representative of 2 experiments, each from a pool of 28–30 embryos with values presented as mean ± SEM of triplicate samples.

To determine whether M2 macrophages would be sufficient to recapitulate *in vitro* the NCAM^+^ phenotype observed *in vivo*, embryonic pancreatic explants depleted of their mesenchymal component, were incubated with macrophages in organ cultures. Macrophages used in these co-cultures were differentiated from mouse BM using M-CSF and polarized toward either M1 or M2 immune functions by cytokine treatment [29]. After three days, epithelial cells within these organ cultures were analyzed by flow cytometry. Two color immunostaining for NCAM and the pan-leukocyte marker CD45, followed by gating on the CD45 negative epithelial population, showed that co-culture with M2 polarized macrophages increases the proportion of NCAM^+^ cells as compared to co-cultures with M1 macrophages or cultures in medium alone (Fig. 4A).

Based on the knowledge that NCAM expression is induced in the islet cell lineage as it develops from ductal progenitors, we sought to determine if the observed NCAM induction was modulated in concert with transcriptional programs regulating islet cell development. Previous reports indicated a role of the transcription factor PAX6 in the regulation of NCAM in neuro-endocrine tissues [43], [44], as well as in the development of the islet cell lineage [45]–[48]. Hence, we set out to analyze expression of PAX6 transcripts in the co-cultures. For this purpose, we used either primers specific for the paired (PD) isoforms of PAX6 (i.e. canonical and 5a variants) [49], [50], or the paired-less ΔPD-PAX6, a variant described as selectively expressed in both developing neural and pancreatic tissues [51]–[53]. We found that while transcription of paired-PAX6 mRNAs is not differentially modulated by M1 *versus* M2 macrophages, the ΔPD-PAX6-specific transcript is significantly up-regulated by about two-folds in M2-macrophages/epithelial cells co-cultures (Fig. 4B). Gene expression of other transcription factors such as NeuroD1, Nkx2.2, Nkx6.1, PDX-1 and Ngn3 was not differentially affected in M2- *versus* M1-marophages/epithelium co-cultures (data not shown).

Further studies were then undertaken to evaluate the expression of ΔPD-PAX6 in the embryonic pancreas. In the developing E14–15.5 pancreas, high levels of E-cadherin and expression of the transcription factor SOX9 characterize pools of ductal progenitors that give rise to both endocrine and exocrine lineages [54], [55], whereas relatively low levels of E-cadherin is observed in epithelial cells already committed to either fates [21]. To determine whether PD and ΔPD-PAX6 transcripts were differentially expressed in these epithelial compartments, single cell suspensions of E14–E15.5 fetal pancreata were sorted as E-cadherin high, intermediate and low populations (Fig. 4C) and mRNA transcription assessed by real time PCR. This analysis revealed that levels of ΔPD-PAX6 transcripts are higher in the epithelial E-cadh^High^ as compared to the E-cadh^Int^ or E-cadh^Low^ fractions (Fig. 4D), indicating preferential expression of this transcript in ductal pools. Control PCRs for E-cadherin- and SOX9-specific transcripts in the sorted populations demonstrated progressive increase of these mRNAs from E-cadh^Low^ to E-cadh^High^ subsets (Fig. 4E), validating the cell subset enrichment obtained by sorting.

**Figure 5:**
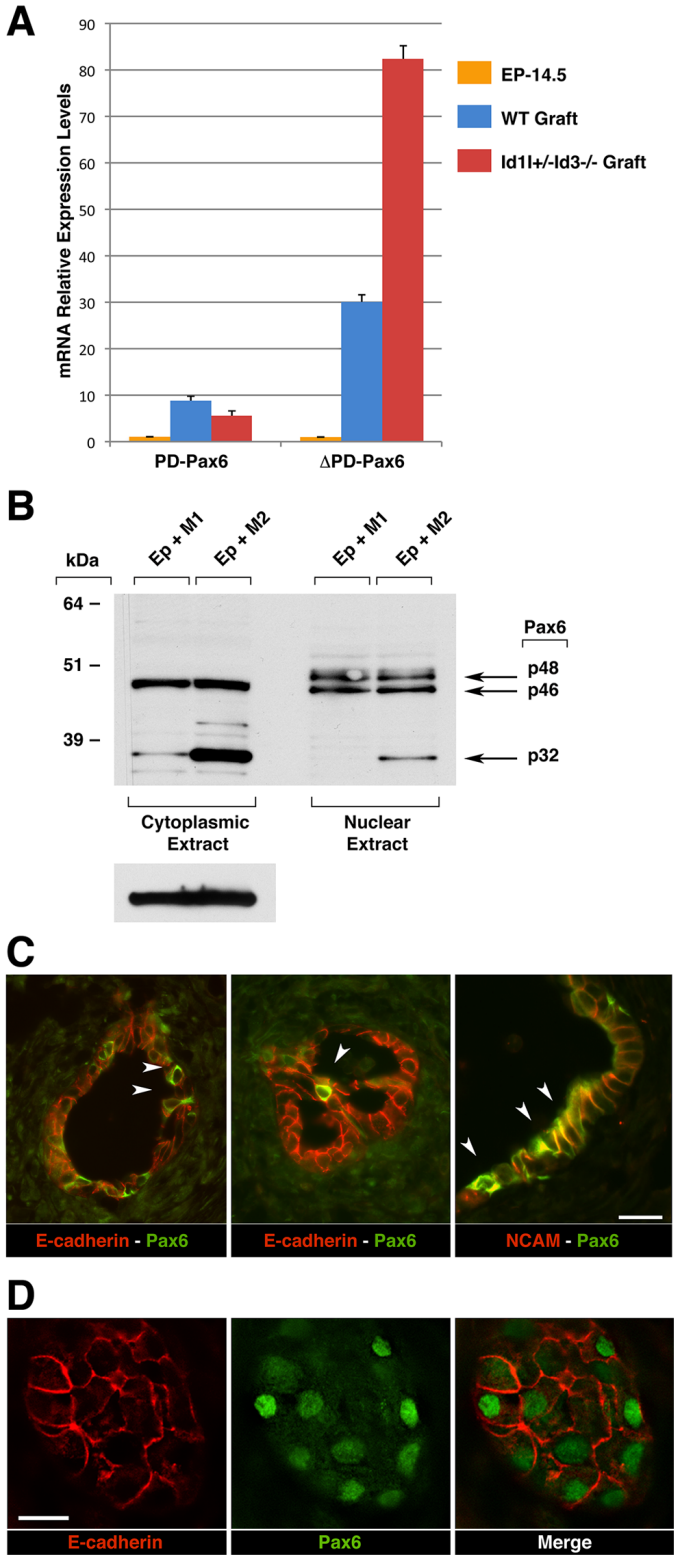
Expression of PAX6 transcripts and protein isoforms in the grafts and cultures of embryonic pancreatic epithelium. (A) qPCR analysis of paired (PD) and paired-less (ΔPD) PAX6 transcripts detected in epithelial grafts from WT or Id1+/−Id3−/− hosts 2 weeks post-transplant, as compared to those detected in epithelial clusters isolated from E14.5 pancreata. Bars represent mean ± SEM of triplicate values normalized to E-cadherin mRNA. Representative of n = 2 experiments. (B) Western blotting analysis of cytoplasmic and nuclear cell lysates from organ cultures of E14.5–15.5 pancreatic epithelium with M1 (EP+M1) or M2 (EP+M2) macrophages. Membranes were probed with an anti-PAX6 antibody or with an anti-β-actin antibody, as loading control. Representative of n = 3 experiments. (C–D) Tissue sections of E14–15.5 pancreatic grafts transplanted in BM-reconstituted Id1+/−Id3−/− mice, immuno-stained for PAX6 (green) and either E-cadherin or NCAM (red). Scale bars = 25 µm in C and 15 µm in D. (C) PAX6 immunoreactivity in ductal structures is observed in individual E-cadherin^+^ cells, within NCAM^+^ ductal domains and with a prevalent cytoplasmic pattern (arrowheads). (D) In contrast, PAX6 immunoreactivity in islet-like cell clusters shows a predominant nuclear localization.

To investigate whether ΔPD-PAX6 induced in organ cultures by M2 macrophages was also differentially expressed *in vivo* in M2-macrophages-enriched grafts of BM-reconstituted Id1+/−Id3−/− mice *versus* WT controls, the grafts were micro-dissected, lysed and their mRNA was analyzed by real time PCR using primers specific for either PD or ΔPD-PAX6 transcripts. In these experiments, to account for the epithelial component of the grafts, levels of PAX6 transcripts were normalized to E-cadherin mRNA and expressed as fold changes over those detected in freshly isolated epithelial clusters from E14–E15 pancreata. This analysis revealed substantial up-regulation of ΔPD-PAX6 over PD-PAX6 transcripts in the grafts of both hosts (Fig. 5A). Levels of ΔPD-PAX6 up-regulation, however, were most dramatic in the grafts of BM-reconstituted Id1+/−Id3−/− mice (i.e. 80 fold induction) as compared to the grafts of WT mice (i.e. 30 fold induction) (Fig. 5A).

Paired canonical and 5a variants of PAX6 transcripts are translated into 46 and 48 kDa proteins, respectively, whereas ΔPD-PAX6 mRNAs have been described to encode for 30–32 kDa proteins [49]–[53], herein referred to as p46, p48 and p32. To determine whether ΔPD-PAX6 transcripts induced by M2 macrophages in organ cultures were translated into protein, and investigate the nuclear *versus* cytoplasm subcellular localization of this protein relatively to other PAX6 isoforms, nuclear and cytoplasmic lysates of the organ cultures were analyzed by Western blotting using an anti-PAX6 antibody specific for a COOH terminal peptide common to all PAX6 isoforms. Consistent with the transcript analysis (Fig. 4B), these experiments confirmed that M2 macrophages increase the expression of a p32 form of PAX6 in pancreatic epithelial organ cultures relative to their M1 counterpart (Fig. 5B). Intriguingly, expression of the p32 isoform was not confined to the nucleus; rather, a considerable amount was retained in the cytoplasm. In addition, induction of this isoform was not observed in the presence of M2 macrophage-conditioned media alone (not shown), indicating the requirement of cell-cell interactions. The paired canonical and 5a isoforms of PAX6 were also detected, but their expression levels were not differentially modulated in the presence of M1 and M2 macrophages (Fig. 5B).

The unusually high cytoplasmic localization of p32 PAX6 induced in organ cultures by M2-macrophages prompted us to investigate whether PAX6 cytoplasmic immunoreactivity identifies selected populations of epithelial cells within the grafts. Indeed two-color immunofluorescence for E-cadherin and PAX6 revealed the presence of scattered single cells with a strong PAX6-specific cytoplasmic immunoreactivity in ductal structures (Fig. 5C, left two panels). Ductal cells with this unusual PAX6 expression pattern were frequently comprised within groups of NCAM^+^ cells (Fig. 5C, right panel) indicating that these two populations were partially overlapping. Unlike the cytoplasmic expression pattern of this unique ductal population, PAX6 immunoreactivity in the islet clusters away from the ducts was predominantly nuclear (Fig. 5D)

Taken together, these results indicate that M2 macrophages recapitulate in organ cultures the induction of NCAM observed in the Id1/Id3 KO transplantation model. They further identify the ΔPD-PAX6 isoform as a hallmark of M2 macrophage inductive functions on the developing pancreatic epithelium.

### NCAM induction in ductal epithelia by M2 macrophages marks cells with a highly migratory phenotype

The enhanced expression of NCAM and ΔPD-PAX6 induced in embryonic pancreatic epithelia by M2-macrophages raised the question as to the functional significance of these cellular responses.

**Figure 6:**
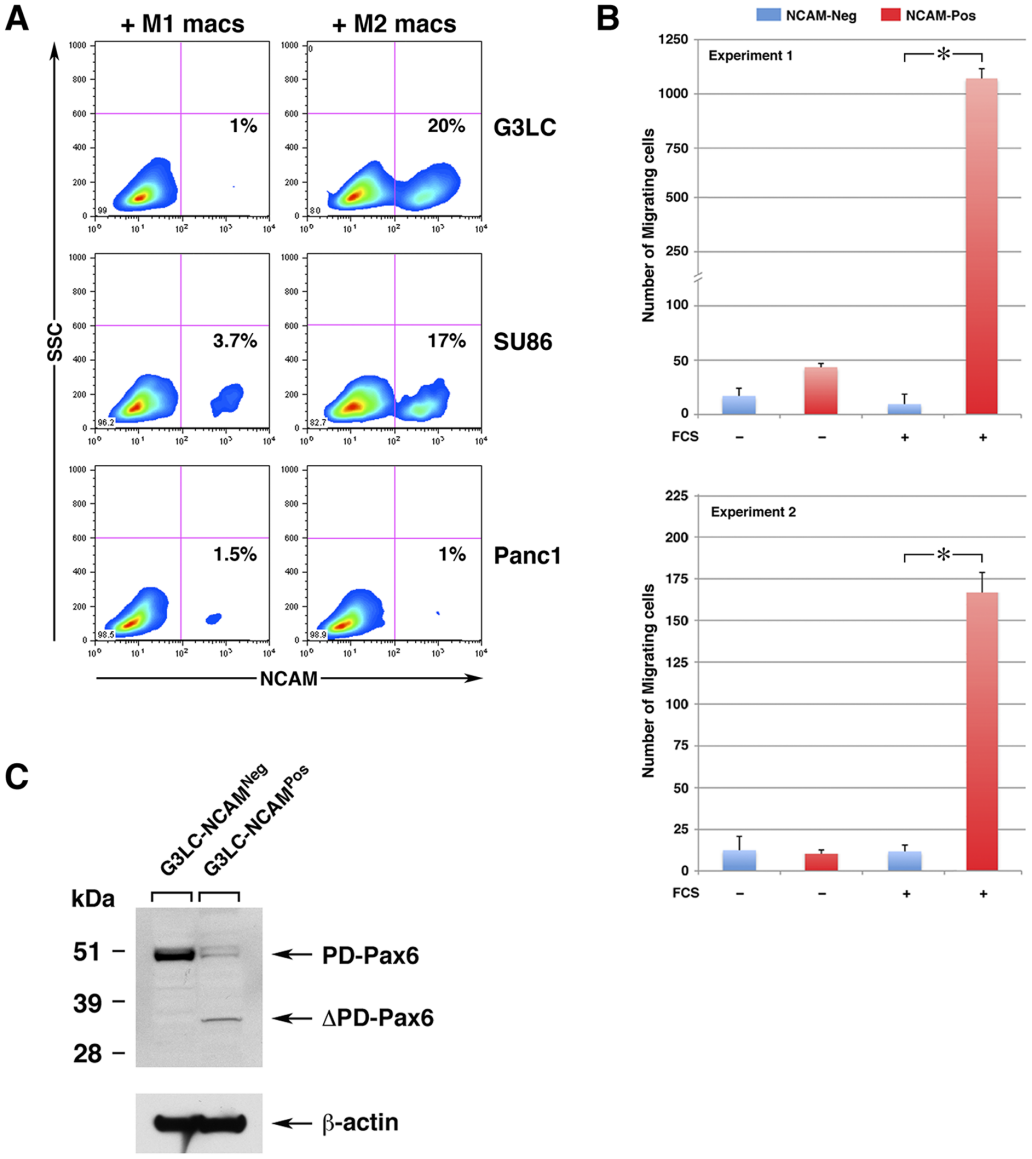
Induction of NCAM and ΔPD-PAX6 by M2 macrophages are overlapping phenotypes associated with enhanced migration. (A) Flow cytometric analysis of G3LC, SU86 and PANC-1 cell lines co-cultured with either M1 (M1 macs) or M2 (M2 macs) macrophages, immuno-stained for NCAM and the pan-leukocyte marker CD45. The contour plots shows cells gated as CD45^neg^ (i.e. epithelial cells only), and the percentage of NCAM^+^ cells are indicated in the right lower quadrant. (B) Migration of G3LC cells sorted from co-cultures with M2 macrophages as CD45^neg^NCAM^Neg^ and CD45^neg^NCAM^Pos^ subsets, in response to 20% FCS. Bars represent the mean ± SD of duplicate samples in each experiment. *p<0.001. (C) Western blotting analysis of PAX6 isoforms expressed in cytoplasmic cell lysates from sorted NCAM^Neg^ and NCAM^Pos^ G3LC cells derived from the M2 macrophages co-cultures shown in panel A.

Over-expression of NCAM in epithelia has been associated with the acquisition of cell motility [56]–[58]. To investigate whether NCAM expression induced by cell-cell interactions with M2 macrophages imparts similar functions on pancreatic ductal epithelia, we turned to ductal epithelial cell lines as a model system. Co-culture of M2 but not M1 macrophages with the pancreatic lines SU86 and G3LC induced NCAM expression on a small, but discrete, subset of cells (Fig. 6A). Hence, in this model system, M2 macrophages recapitulate the induction of NCAM expression observed with the pancreatic embryonic epithelium in organ cultures and *in vivo*. Nevertheless, competence to generate this cell subset was line-specific, since the PANC-1 line did not show the emergence of a significant number of NCAM^+^ cells. As for ΔPD-PAX6, M2 macrophage conditioned media alone did not induce NCAM expression in neither lines (not shown). Although the precise mechanism of NCAM protein up-regulation remains to be fully elucidated, qPCR analysis of epithelial cells co-cultured with M2 macrophages did not reveal increased expression of NCAM-specific transcripts as compared to the levels detected in the parental lines (Figure S2), suggesting a post-transcriptional regulation of NCAM expression.

To determine whether NCAM^+^ G3LC ductal cells generated in co-culture with M2 macrophages had acquired an enhanced migratory phenotype, cell cultures were labeled for CD45 and NCAM and sorted as CD45^Neg^NCAM^Pos^ and CD45^Neg^NCAM^Neg^. The two subsets were then used in chemotactic migration assays. These experiments revealed that NCAM^Pos^ cells exhibited significantly higher migratory responses to chemotactic stimuli provided by FCS than their NCAM^Neg^ counterparts (Fig. 6B,). Further Western blotting analysis of cell lysates from the sorted populations, revealed that the NCAM^Pos^ but not the NCAM^Neg^ cells expressed a 32 kDa protein specifically detected by the anti-PAX6 antibody, consistent with the expression of a ΔPD-PAX6 isoform within the highly migratory NCAM^Pos^ subset (Fig. 6C). PD-PAX6 was also down-regulated in this latter fraction.

These results show that induction of NCAM on the ductal epithelium by functionally polarized M2 macrophages selects for cells with enhanced competency to respond to exogenous migratory cues. The data further show that NCAM and ΔPD-PAX6 are induced within the same cell population.

### Low levels of expression of ΔPD-PAX6 are sufficient to impart a migratory phenotype in pancreatic ductal epithelia

The *in vivo* and *in vitro* studies presented above indicate that induction of NCAM and ΔPD-PAX6 by M2-macrophages are overlapping phenotypes, suggesting a possible functional relationship of NCAM and ΔPD-PAX6 expression. To date, no biological functions have been attributed to the ΔPD-PAX6 isoform.

**Figure 7:**
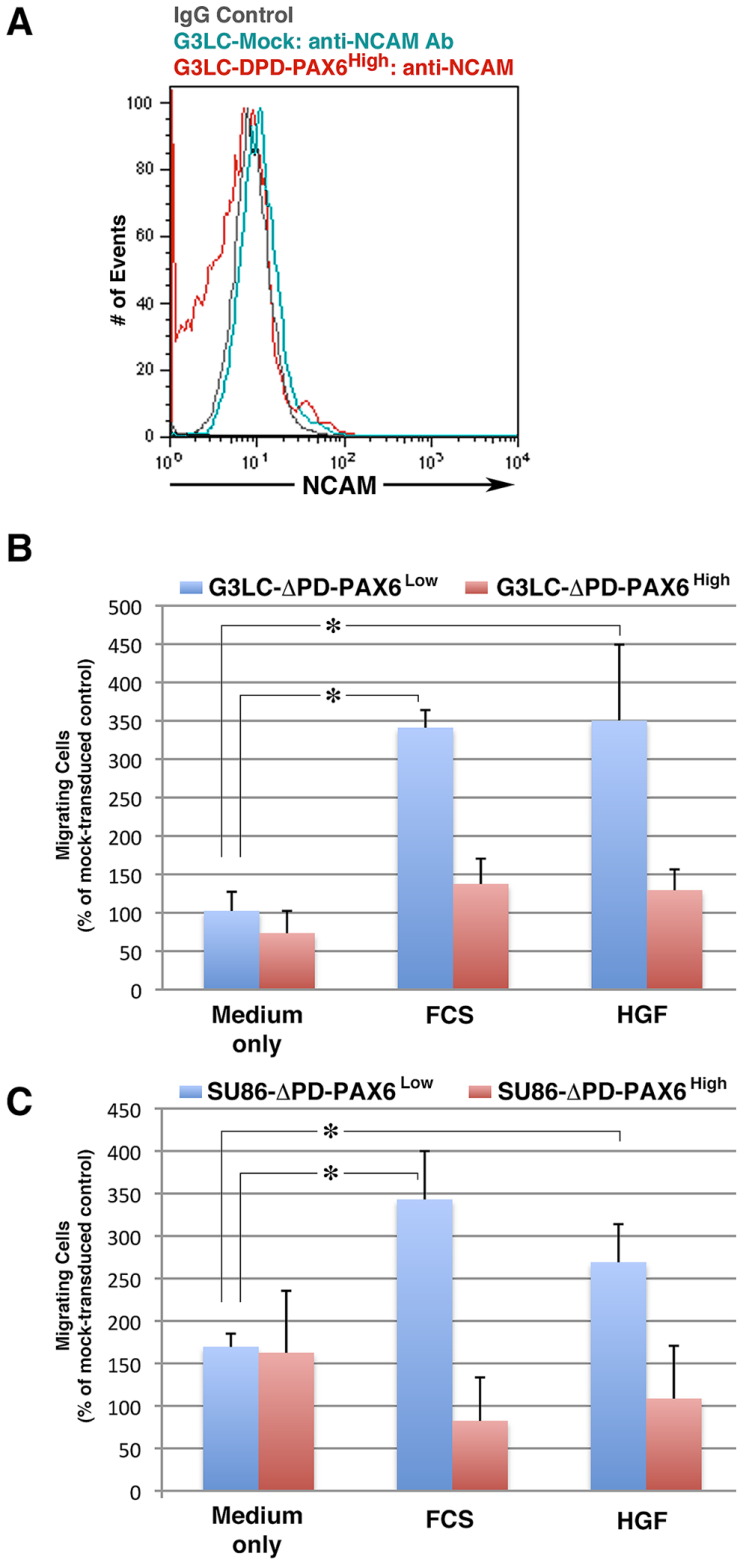
Over-expression of ΔPD-PAX6 enhances migration of pancreatic epithelial cells but does not induce NCAM. (A) Flow cytometric analysis of G3LC cells transduced with either empty (G3LC-Mock) or ΔPD-PAX6 expressing lenti-viruses at 30 MOI (G3LC-ΔPD-PAX6^High^) immunostained for NCAM. Over-expression of ΔPD-PAX6 is not sufficient to induce NCAM expression. (B–C) Migratory responses of Low (ΔPD-PAX6^Low^) and High (ΔPD-PAX6^High^) ΔPD-PAX6 expressors, generated by lentivirus transduction at 5 and 30 MOI, respectively, in response to chemotactic stimuli provided by FCS and HGF/SF as compared to medium alone. Bars represent mean ± SEM of n = 3 experiments. * *p*≤0.001.

To shed light into the functions mediated by this PAX6 isoform, we amplified this variant mRNA from mouse fetal brain cDNA using PCR primers spanning the exon3/8 boundary and the 3′ UTR of PAX6, as described [52], and cloned the resulting cDNA product into a lenti-viral vector carrying constitutive expression of a GFP reporter. Viral transduction of HEK-293T or G3LC cells and Western blotting analysis of cell protein extracts confirmed that the cloned cDNA is expressed in epithelial cells as a stable protein of ∼32 kDa, with both a nuclear and cytoplasmic localization (Figure S3). Following these experiments, G3LC and SU86 cells were infected at 5 and 30 MOI, and transduced GFP^+^ cells were sorted by flow cytometry to generate ΔPD-PAX6^Low^ and ΔPD-PAX6^High^ sub-lines. Phenotypic characterization of these lines demonstrated that expression of ΔPD-PAX6 at the levels achieved in these transduction experiments does not induce NCAM (Fig. 7A). However, ΔPD-PAX6^Low^ but not ΔPD-PAX6^High^ lines exhibited enhanced migratory responses to chemotactic stimuli provided by either FCS or HGF/SF as compared to mock-transduced control lines (Fig. 7B–C).

These results demonstrate that the ΔPD-PAX6 isoform does not influence NCAM protein expression in ductal epithelia; however, at low levels, it is sufficient to increase cell motility. Collectively, the data identify induction of NCAM and regulation of ΔPD-PAX6 expression as two distinct phenotypes elicited by M2 macrophages, which co-operate to enhance cell motility in ductal epithelial cells.

### Over-expression of ΔPD-PAX6 modulates epithelial cell cycle progression in a dose dependent fashion and is associated with the acquisition of islet progenitor phenotypes

**Figure 8:**
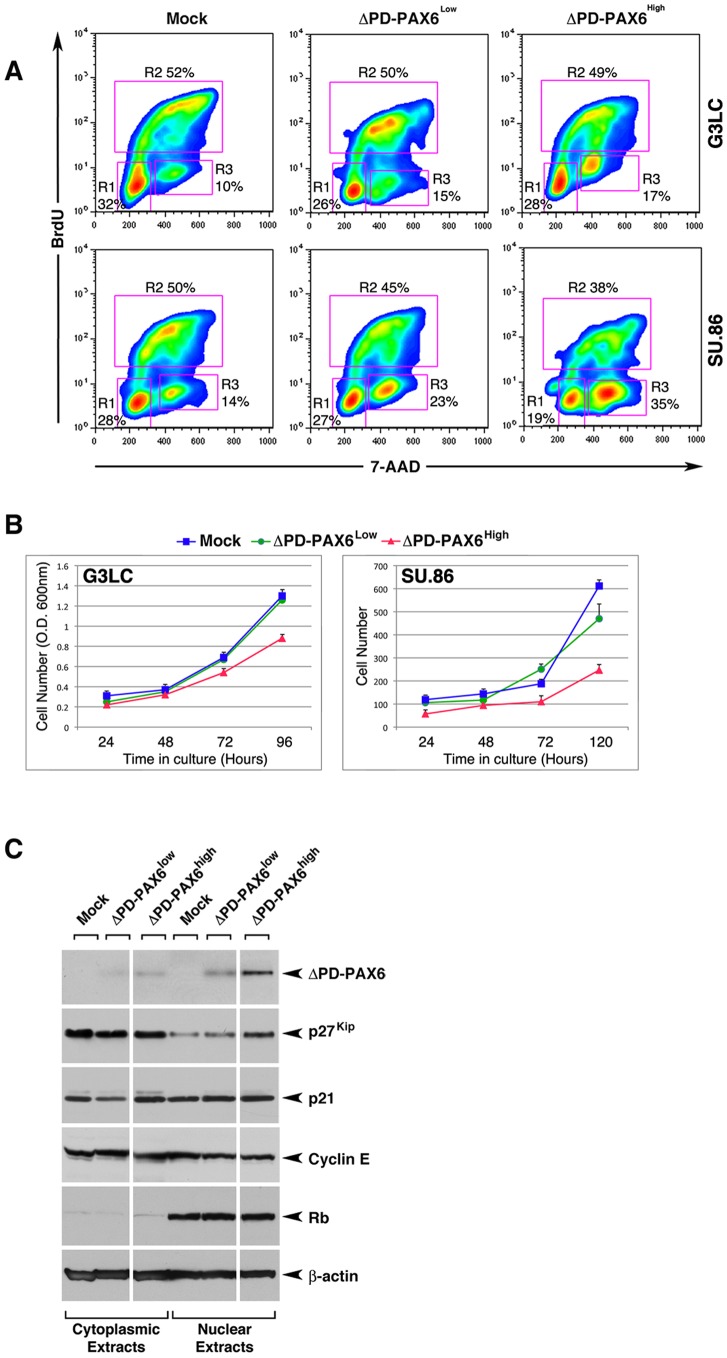
Modulation of epithelial cell cycle progression by ΔPD-PAX6. (A) Cell cycle analysis of mock-transduced, Low (ΔPD-PAX6^Low^) and High (ΔPD-PAX6^High^) ΔPD-PAX6 G3LC, and SU.86 cells, as measured by flow cytometric analysis of DNA content and BrdU incorporation. In each plot, percentages of cells in G0/G1, S and G2/M are indicated in regions R1, R2 and R3 respectively. Representative of n = 2 experiments. (B) Growth curves of G3LC and SU86 lines as a function of ΔPD-PAX6 expression levels. Representative of n = 3 experiments. (C) Western blotting analysis of PAX6, p27^kip^, p21^cip^, cyclin E, Retinoblastoma (Rb) protein and β-actin expressed in cell lysates obtained from Mock, Low (ΔPD-PAX6^Low^) and High ΔPD-PAX6 (ΔPD-PAX6^High^) G3LC. Representative of n = 2 experiments.

Another effect we have observed in the M2 macrophage-enriched transplantation model is a negative regulation of epithelial cell proliferation in islet cell clusters, as measured by a decreased frequency of Insulin^+^PCNA^+^ cells (Fig. 2D). To investigate whether induction of ΔPD-PAX6 alone in pancreatic epithelial cells could influence cell cycle progression, we performed cell cycle analysis of ΔPD-PAX6^Low^ and ΔPD-PAX6^High^ lines. This analysis revealed that transduction of ΔPD-PAX6 results in a decreased proportion of cells in S phase and accumulation of cells in G2/M phase (Fig. 8A, Regions 2 and 3, respectively) indicating a blockage at this latter checkpoint of the cell cycle. Notably, this effect on cell cycle progression was most dramatic in the ΔPD-PAX6^High^ lines, indicating a dosage dependent effect of ΔPD-PAX6. Accordingly, growth curves of either ΔPD-PAX6^High^ SU86 and G3LC lines demonstrated a ∼50% and 30% decrease in cell numbers, respectively, at 5 days of culture (Fig. 8B). Further Western blotting analysis of modulators of the cell cycle demonstrate that expression of ΔPD-PAX6 is associated with up-regulation of the cell cycle brake p27^kip^ in the nuclear fraction, in a fashion proportional to the levels of ΔPD-PAX6 expression. In contrast no significant changes in the expression levels and/or sub-cellular distribution of p21^cip^ and cyclin E were detected (Fig. 8C). Based on these results, we investigated the pattern of expression of p27^kip^ in the embryonic pancreatic transplants. These studies revealed that in WT hosts p27^kip^-specific immunoreactivity is predominantly localized in the cytoplasm, with few scattered cells exhibiting nuclear staining, whereas grafts in Id1/Id3-deficient hosts displayed both cytoplasmic and nuclear immunoreactivity (Figure S4).

We further analyzed ΔPD-PAX6-transduced G3LC and SU.86 lines for the expression of markers of islet lineages (e.g. NKX6.1, NKX2.2, PDX-1, Ngn3, MAF-B) and pancreatic hormones (e.g. insulin, glucagon, somatostatin and PP). PCR analysis of these transcripts in the linear range of amplification revealed that ΔPD-PAX6 transduction in these lines does not result in detectable expression of islet hormones (Figure S5). However, these studies showed that both G3LC ΔPD-PAX6^Low^ and ΔPD-PAX6^High^ cells up-regulate the expression of transcription factors activated during islet cell lineage development, i.e. NKX6.1, NKX2.2, PDX-1 and MAF-B. NKX6.1 and MAF-B, but not NKX2.2, were also up-regulated in SU86 ΔPD-PAX6^High^ cells as compared to the parental line (Figure S5).

Taken together, these results provide novel evidence for a functional link between modulation of ΔPD-PAX6 and activation of cellular pathways involved in epithelial cell growth arrest. They further indicate the potential of ΔPD-PAX6 to positively regulate the progression of pancreatic ductal epithelia toward endocrine progenitor cell fates.

## Discussion

In the developing pancreas, the migration of epithelial progenitors from ductal compartments into the surrounding mesenchyme and its coordination with differentiative events are thought to be critical to the development of both the exocrine and the endocrine compartments of this organ. Enhanced progenitor delamination and islet neogenesis from putative duct associated precursors have also been invoked in models of pancreatic tissue regeneration associated with inflammation [37]–[42]. To date, the possible role of macrophages and their functional polarization in these phenomena has remained virtually unexplored.

We found that M2 macrophages/epithelial interactions *in vivo* and *in vitro* result in the expression of NCAM in a subpopulation of ductal cells and that NCAM induction marks cells with migratory capabilities far greater than the NCAM-negative ductal pool. Previous reports have demonstrated a role of NCAM in cell types segregation and tissue patterning during embryogenesis, and shown that its induction in epithelial cells may act as a switch of cell migration by disrupting cadherin-mediated cell adhesion and/or regulating FGF receptor signaling [56]–[58]. Collectively, these observations suggest that macrophages may be involved in modulating epithelial cell migration not only by remodeling the extracellular environment, as previously described [18], but also by directly regulating the expression of select motogenic adhesion receptors such as NCAM in the epithelium itself. This migratory phenotype may be relevant to the segregation of islet progenitors from ductal domains. Consistent with this possibility, we observed that the NCAM^+^ ductal epithelium comprises PDX-1^+^ and insulin^+^ cells, a pattern previously described in models of islet neogenesis [37]–[42].

In searching for putative inductive functions of macrophages on pancreatic transcriptional programs [19]–[21], [59]–[65], we found that the interaction of embryonic pancreatic epithelium with M2 macrophages *in vitro* and *in vivo* results in the up-regulation of a truncated isoform of PAX6. Previous studies have shown that activation of the PAX6 locus in vertebrates results in three main PAX6 transcripts encoding distinct proteins with variations in their functional domains. These include the “canonical” and the “5a variant” of PAX6, both containing a “paired” (PD) and a “paired-type” homeodomain for DNA binding, as well as a “paired-less” or ΔPD-PAX6 variant lacking the paired domain but maintaining the homeodomain [49]–[53]. Studies in animal models bearing mutations that disrupt the expression of PD-PAX6 isoforms have shown their critical role in modulating the balance of developing glucagon- *versus* insulin-producing islet cells, as well the expression of genes required for endocrine secretory functions [45]–[48]. Much less is known on how expression of ΔPD-PAX6 is regulated and what its function(s) might be. Previous studies have reported a spatially and temporally restricted expression of this PAX6 variant in the embryonic retinas, olfactory epithelium and pancreas [51], [66], [67], suggesting functions linked to the development of these tissues [68]. Our data provide novel evidence that induction of ΔPD-PAX6 in the embryonic pancreatic epithelium occurs upon interaction with M2 macrophages, uncovering one potential cellular mechanism regulating its expression. We further show significant up-regulation of ΔPD-PAX6 during tissue healing, suggesting that this isoform may be implicated not only in developmental events, but also in repair responses of this tissue.

**Figure 9:**
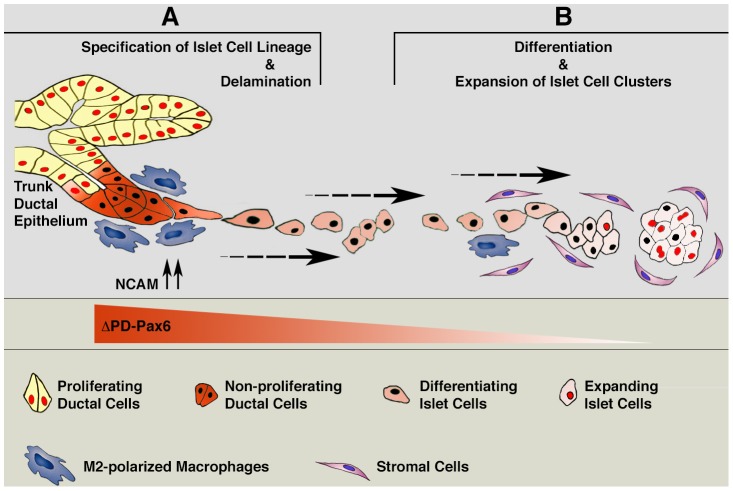
Hypothetical model of macrophages-induced developmental effects on embryonic pancreatic epithelium. (A) Interactions of M2 macrophages with proliferating ductal epithelial cells induce expression of NCAM in delaminating progenitors. Concomitantly, high levels of ΔPD-PAX6 are induced. This latter change results in slowing-down the cell cycle, allowing for the initiation of differentiative events in progenitors committed to endocrine lineages. At the same time, NCAM induction, through disruption of E-cadherin and/or potentiation of FGF signaling, facilitates sorting of committed progenitors out of the ductal lining. (B) Motility of the committed progenitors increases as high levels of ΔPD-PAX6 progressively wear off, allowing their delamination and migration away from the ducts. Eventually, expression of ΔPD-PAX6 returns to very low levels in endocrine-committed cells, permitting migration arrest and resumption of proliferation in response to pro-proliferative cues provided by stromal cells, leading to the formation of islet cell clusters. We suggest that regulation of such ΔPD-PAX6-driven functions, reliant on cell-cell interactions with M2 macrophages and exquisitely sensitive to gene dosage, provides a novel mechanism explaining how cell cycle withdrawal and initiation of cell migration may be functional linked and coordinated in epithelial cells committed to leave ductal progenitor pools.

We found that expression of ΔPD-PAX6 transcripts is enriched in ductal epithelia of the fetal pancreas. It was reported that the PAX6 gene locus can be activated in the primitive pancreatic duct at E9.5 [45], [47], [69], and in scattered duct-associated epithelial cells in models of islet neogenesis [39]. Nevertheless, our finding of a ΔPD-PAX6 variant in embryonic ductal cells uncovers a previously unrecognized complexity in patterns of PAX6 isoforms expressed in distinct pancreatic populations. Importantly, the proposed role of embryonic ductal compartments as source of multipotent progenitors [70] implies that ΔPD-PAX6 may function, at least in part, by regulating early developmental events rather than terminal endocrine maturation, as ascribed to canonical full-length PAX6 [47]. Indeed, we found that over-expression of ΔPD-PAX6 in ductal epithelial cells is associated with up-regulation of early markers of islet cell lineages, including PDX-1, NKX2.2, NKX6.1 and MAF-B. Concomitantly, ΔPD-PAX6 over-expression enhances cell migration and induces cell cycle withdrawal, two functions required for progenitor delamination and differentiation, prior to terminal maturation. Intriguingly, there is a clear gene dosage effect of ΔPD-PAX6 on these cellular responses in such a way that while low levels of expression are permissive for enhancing cell migration, high levels are most effective at inducing cell cycle exit. These results suggest a step-wise model (Fig. 9) by which cell-cell interactions of proliferating ductal epithelial cells with M2 macrophages results initially in induction of NCAM and high levels of ΔPD-PAX6 expression, thereby providing signals for both facilitating sorting out from the ductal compartment and exiting the cell cycle. Thereafter, as levels of ΔPD-PAX6 decline, delamination and migration of such progenitors into the surrounding mesenchyme is promoted. Ultimately, release from M2 macrophages interactions triggering ΔPD-PAX6 up-regulation may be required for endocrine-committed progenitors to resume cell proliferation away from the ducts in response to other mesenchymal cues and expand into islet cell clusters [22], [24]. This expectation is consistent with our results in the Id1/Id3 deficient mouse, showing that embryonic epithelial transplants exposed to a strong M2 macrophage inflammatory milieu exhibit enhanced frequency of endocrine committed cells emerging from the ducts, but decreased proliferation of their progeny in the islet clusters.

The mechanisms underlying enhanced cell migration and slow down of the cell cycle induced by ΔPD-PAX6 warrant further studies. This is a complex undertaking because there is evidence that, depending on the cellular context, ΔPD-PAX6 may either enhance or repress several pathways by establishing inter-molecular interactions with other transcriptional regulators [52], [71]–[74]. These include binding to regulators of the cell cycle (e.g. retinoblastoma) as well as to other PAX6 isoforms [72], [73]. In this regard, it is noteworthy that some target genes of canonical PAX6 itself include mediators of cell migration [75], [76], and that developmental effects of canonical PAX6 in neuronal lineages are also gene-dosage dependent [77]. Hence it is possible that ΔPD-PAX6 acts as cell-context dependent modulator of distinct effectors to coordinate epithelial cell cycle withdrawal, migration, and differentiation. Beyond its relevance to embryonic development, the observed effects of ΔPD-PAX6 induction on cell cycle progression in pancreatic cell lines identify a mechanism potentially critical to the control of pathological tissue growth, such as pancreatic tumor progression. Given the fact that the PAX6 locus can be abnormally activated in tumors of many branched epithelial organs [75], [78], this finding may have implications for the regulation of cell growth in other tumor cell types as well.

Previous reports have provided evidence pro-proliferative effects of pancreatic fibroblasts on the developing epithelium [22], [24]. Our finding that M2 macrophages, through up-regulation of ΔPD-PAX6, may actually slow down the progression of pancreatic epithelia through the cell cycle, suggests that similarly polarized macrophages populating the developing pancreas could function to counter-regulate mesenchymal pro-proliferative cues. This is further supported by the observation that the blockade in cell cycle progression is accompanied by increased nuclear localization of p27^kip^, a cyclin-dependent kinase inhibitor shown to be expressed in pancreatic epithelia that have exited the cell cycle and committed to differentiation [79], [80]. Our results are also consistent with the observation that pancreatic tissue developing in the absence of macrophages exhibits an increased proliferation of ductal compartments and a decreased endocrine cell mass [25], [26]. Taken together, these observations are consistent with functionally opposing effects of M2 macrophages and mesenchymal fibroblasts on cell cycle progression of pancreatic epithelia, and argue that in the developing pancreas, a spatially restricted positioning and/or activation of M2 macrophages must be present to allow for their anti-proliferative effects to take place on progenitors delaminating from the ductal compartment, but not on their islet cell progeny.

Collectively, our studies uncover a novel role of macrophages in their M2 state of activation as positive regulators of pancreatic progenitors recruitment and differentiation toward the islet cell lineage, as well as modulators of islet cell cycle progression. Although the molecular effectors of these macrophage-driven functions remain to be identified, our findings point to a cellular mechanism that could be exploited in pancreatic tissue regeneration. Hence, in light of these results it may be important to test whether brakes on islet regenerative responses *in vivo* following injury and/or degeneration may be modulated by targeting the anti-proliferative effects of M2 macrophages. In addition, the functional properties of macrophages identified here may be harnessed for the development of improved protocols of *in vitro* directed differentiation of islet cells from either ESC or iPSC preparations [81]–[83]. Hence, macrophages with distinct states of functional activation may be exploited to promote either expansion or differentiation of stem cell/progenitor cell populations.

## Supporting Information

Figure S1 **Frequency of apoptotic cells in grafts of embryonic pancreatic epithelium.** Morphometric analysis of apoptotic cells detected by TUNEL assay within E-cadherin^+^ areas of grafts retrieved from BM-reconstituted WT (blue bar) and Id1/Id3-deficient (red bar) hosts. Bars represent means ± SEM of determinations performed in grafts from WT (n = 3) and Id1/Id3-deficient mice grafts (n = 5). *NS* = not significant.
(TIF)

Figure S2 **NCAM mRNA expression in the NCAM^Pos^ and NCAM^Neg^ fractions of ductal epithelium/macrophages co-cultures.** Real time qPCR analysis of NCAM-specific transcripts detected in G3LC and SU.86 cells sorted as CD45^Neg^NCAM^Neg^ (blue bars) and CD45^Neg^NCAM^Pos^ (red bars) fractions from co-cultures with M2-polarized macrophages. Levels of NCAM transcripts detected in NCAM^Pos^ fractions of either cell lines are not significantly different than those detected in the NCAM^Neg^ fraction, or corresponding parental line (yellow bars). Bars represent means ± SEM of n = 3 sorting experiments using G3LC cells and n = 1 experiment using SU.86 cells, with each experiment run in triplicate samples.
(TIF)

Figure S3 **Transduction of ΔPD-PAX6 in epithelial lines.** Western blotting analysis of detergent lysates from HEK-293T and G3LC cells, transduced with empty (mock) or ΔPD-PAX6 expressing lenti-vectors. Blots were probed with either anti-PAX6 antibody or anti-beta actin antibody, as loading control. In both lines, expression of a 32 kDa PAX6 isoform is detectable in nuclear and cytoplasmic lysates. The G3LC line also expresses endogenous paired PAX6 variants (PD-PAX6).
(TIF)

Figure S4 **Expression pattern of p27^Kip^ in grafts of embryonic pancreatic epithelium.** Tissue sections of E14–15.5 pancreatic epithelial grafts transplanted in BM-reconstituted WT mice (upper panels) or Id1+/−Id3−/− mice (lower panels), stained by two-color immuno-fluorescence for p27*^Kip^* (green) and DAPI (blue). Dotted lines delineate boundaries of islet-like cell clusters. Grafts from WT hosts exhibit a predominant cytoplasmic localization of p27*^Kip^*, whereas grafts from Id1+/−Id3−/− mice reveal both cytoplasmic and nuclear localizations (arrowheads). Scale bar in middle panels = 30 µm; Scale bar in right panels = 5 µm.
(TIF)

Figure S5 **Transduction of ΔPD-PAX6 in pancreatic ductal lines results in the expression of islet lineage phenotypes.** PCR analysis of the indicated transcripts expressed in Mock (Ctrl), ΔPD-PAX6^High^, and ΔPD-PAX6^Low^ G3LC and SU.86 lines. A cDNA prepared from human fetal pancreas (Hu-PANC) was used as a positive control. Representative of n = 2 experiments.
(TIF)
