# Supplementary figures and images for: Macrophage/Epithelium Cross-Talk Regulates Cell Cycle Progression and Migration in Pancreatic Progenitors
Source: PLoS One. 2014 Feb 19;9(2):e89492. doi: 10.1371/journal.pone.0089492 (PMC3929706; doi:10.1371/journal.pone.0089492)

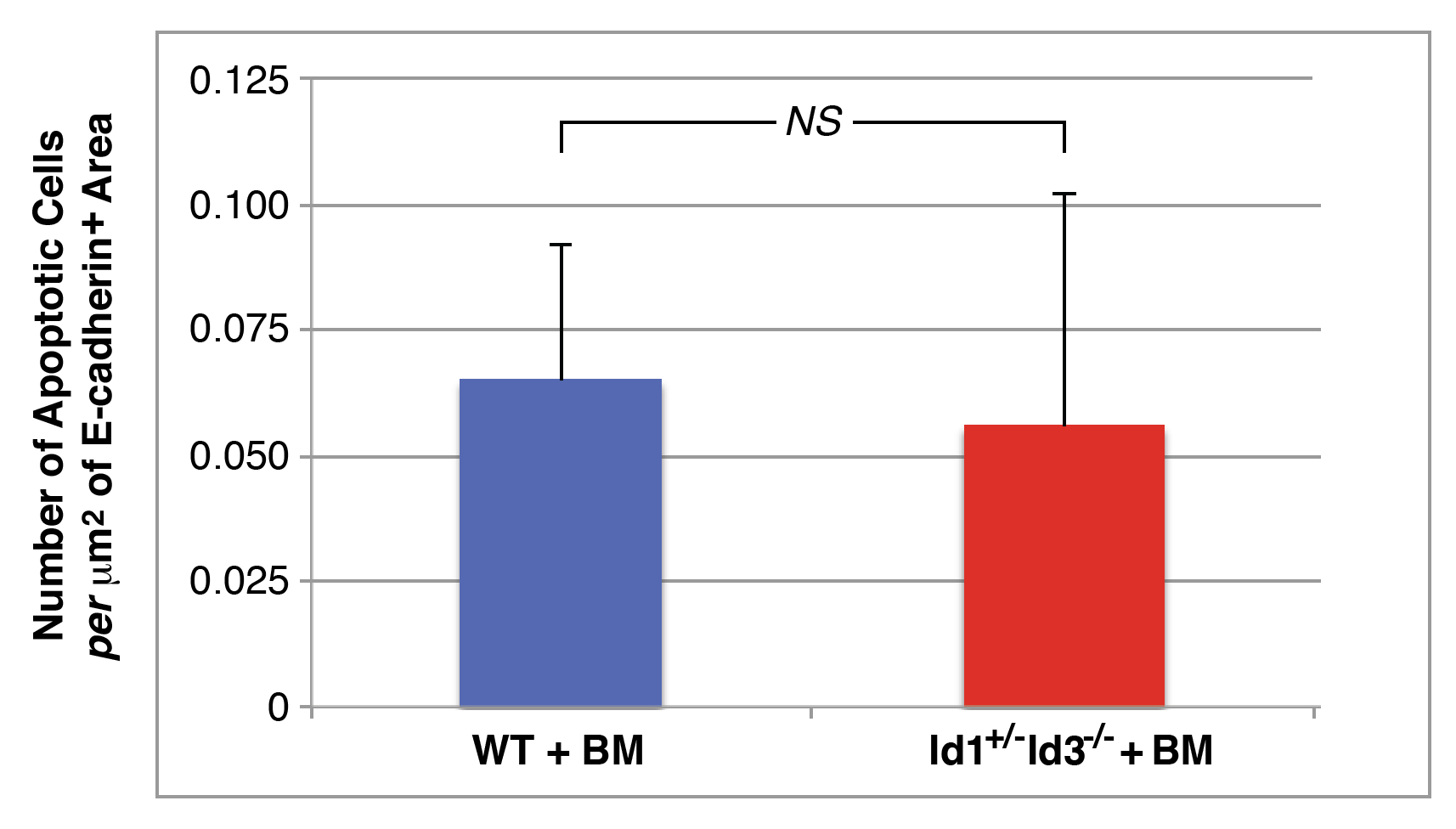

Supplement: Figure S1 — Frequency of apoptotic cells in grafts of embryonic pancreatic epithelium. Morphometric analysis of apoptotic cells detected by TUNEL assay within E-cadherin+ areas of grafts retrieved from BM-reconstituted WT (blue bar) and Id1/Id3-deficient (red bar) hosts. Bars represent means ± SEM of determinations performed in grafts from WT (n = 3) and Id1/Id3-deficient mice grafts (n = 5). NS = not significant. (TIF) [file pone.0089492.s001.tif]

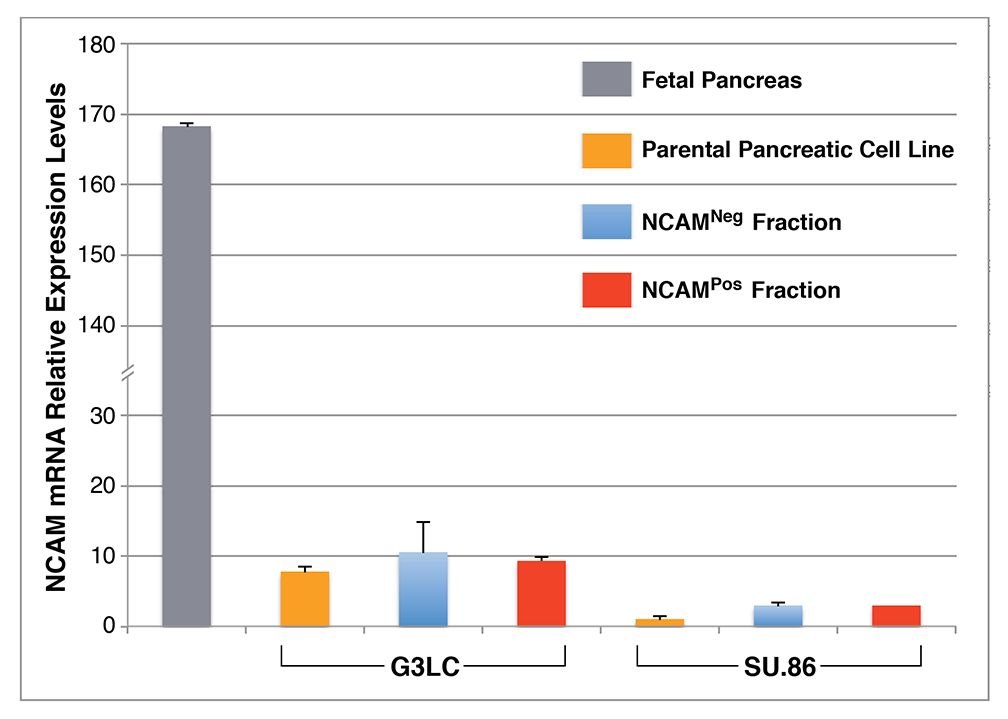

Supplement: Figure S2 — NCAM mRNA expression in the NCAMPos and NCAMNeg fractions of ductal epithelium/macrophages co-cultures. Real time qPCR analysis of NCAM-specific transcripts detected in G3LC and SU.86 cells sorted as CD45NegNCAMNeg (blue bars) and CD45NegNCAMPos (red bars) fractions from co-cultures with M2-polarized macrophages. Levels of NCAM transcripts detected in NCAMPos fractions of either cell lines are not significantly different than those detected in the NCAMNeg fraction, or corresponding parental line (yellow bars). Bars represent means ± SEM of n = 3 sorting experiments using G3LC cells and n = 1 experiment using SU.86 cells, with each experiment run in triplicate samples. (TIF) [file pone.0089492.s002.tif]

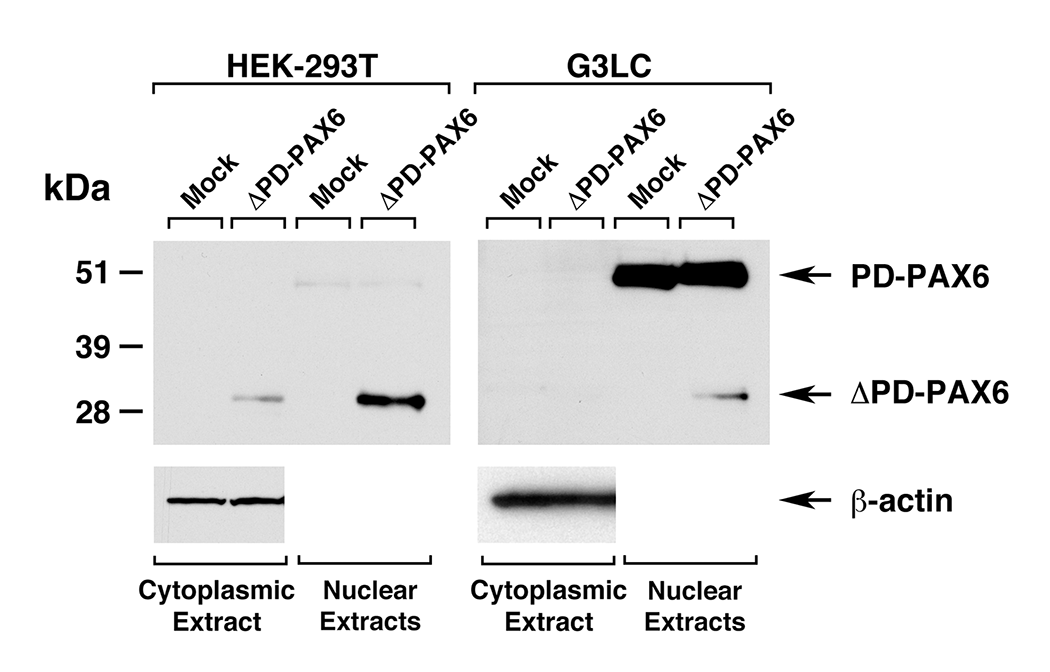

Supplement: Figure S3 — Transduction of ΔPD-PAX6 in epithelial lines. Western blotting analysis of detergent lysates from HEK-293T and G3LC cells, transduced with empty (mock) or ΔPD-PAX6 expressing lenti-vectors. Blots were probed with either anti-PAX6 antibody or anti-beta actin antibody, as loading control. In both lines, expression of a 32 kDa PAX6 isoform is detectable in nuclear and cytoplasmic lysates. The G3LC line also expresses endogenous paired PAX6 variants (PD-PAX6). (TIF) [file pone.0089492.s003.tif]

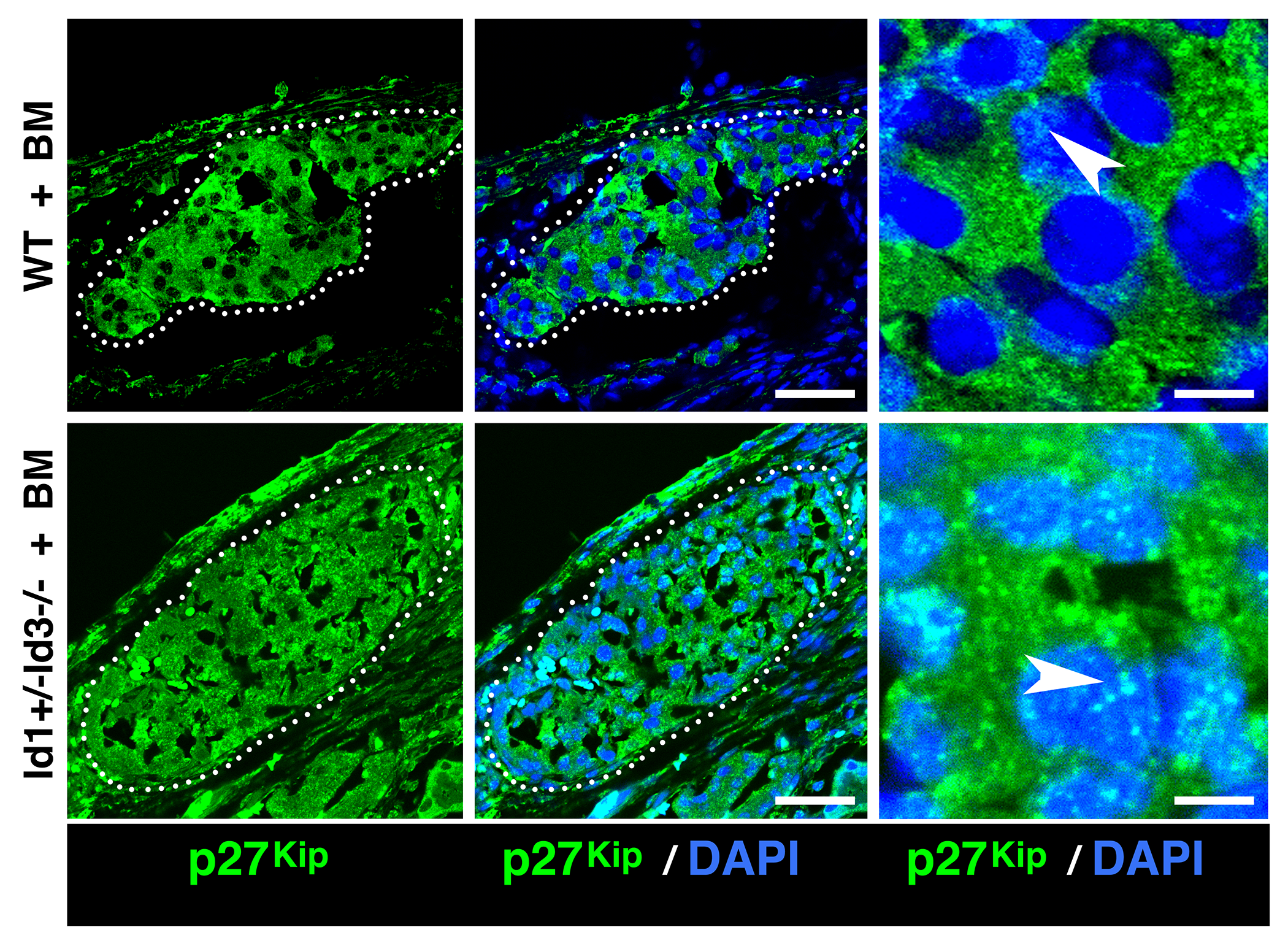

Supplement: Figure S4 — Expression pattern of p27Kip in grafts of embryonic pancreatic epithelium. Tissue sections of E14–15.5 pancreatic epithelial grafts transplanted in BM-reconstituted WT mice (upper panels) or Id1+/−Id3−/− mice (lower panels), stained by two-color immuno-fluorescence for p27Kip (green) and DAPI (blue). Dotted lines delineate boundaries of islet-like cell clusters. Grafts from WT hosts exhibit a predominant cytoplasmic localization of p27Kip, whereas grafts from Id1+/−Id3−/− mice reveal both cytoplasmic and nuclear localizations (arrowheads). Scale bar in middle panels = 30 µm; Scale bar in right panels = 5 µm. (TIF) [file pone.0089492.s004.tif]

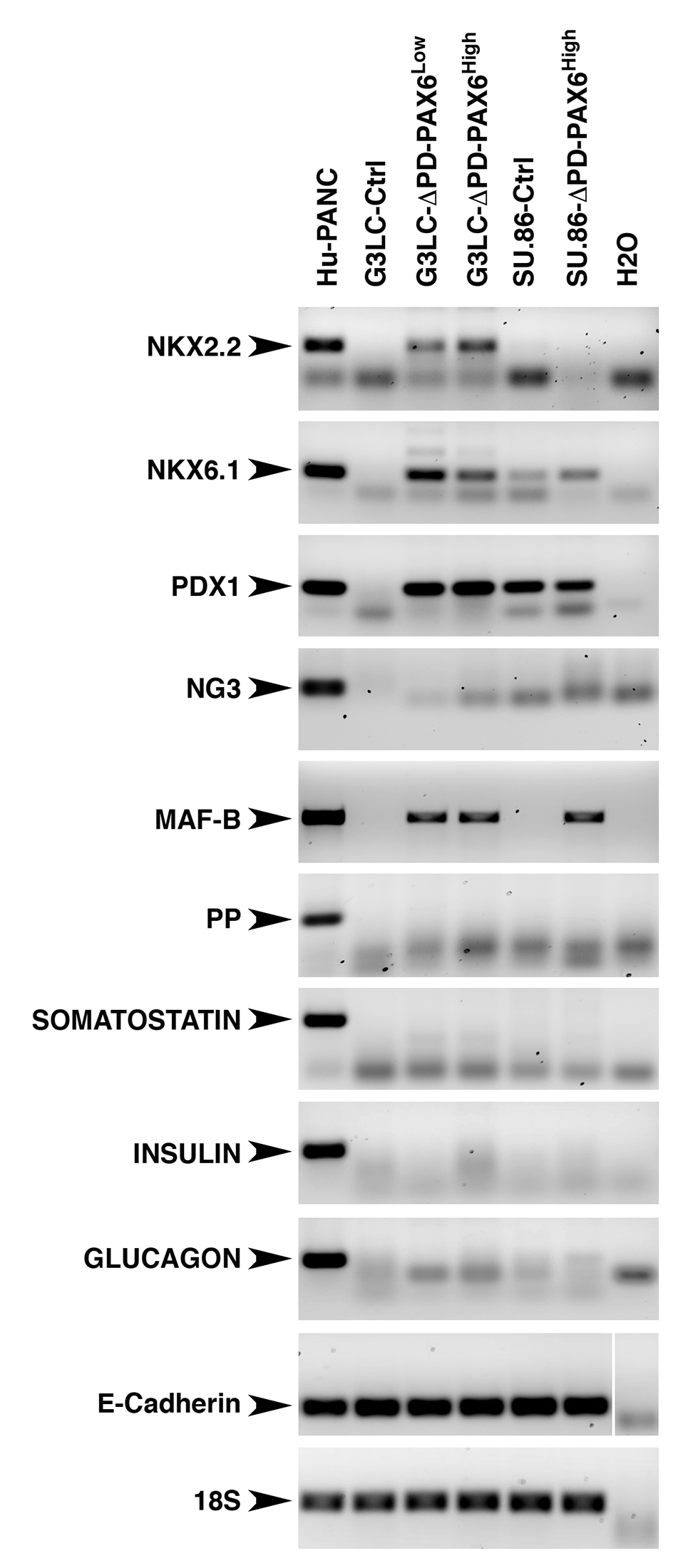

Supplement: Figure S5 — Transduction of ΔPD-PAX6 in pancreatic ductal lines results in the expression of islet lineage phenotypes. PCR analysis of the indicated transcripts expressed in Mock (Ctrl), ΔPD-PAX6High, and ΔPD-PAX6Low G3LC and SU.86 lines. A cDNA prepared from human fetal pancreas (Hu-PANC) was used as a positive control. Representative of n = 2 experiments. (TIF) [file pone.0089492.s005.tif]
